# Control of myeloid cell functions by nociceptors

**DOI:** 10.3389/fimmu.2023.1127571

**Published:** 2023-03-17

**Authors:** Pavel Hanč, Marie-Angèle Messou, Yidi Wang, Ulrich H. von Andrian

**Affiliations:** ^1^ Department of Immunology, Harvard Medical School, Boston, MA, United States; ^2^ The Ragon Institute of Massachusetts General Hospital (MGH), Massachusetts Institute of Technology (MIT) and Harvard, Cambridge, MA, United States

**Keywords:** neuroimmune interactions, nociceptors, myeloid leukocytes, neuropeptides, control of immunity

## Abstract

The immune system has evolved to protect the host from infectious agents, parasites, and tumor growth, and to ensure the maintenance of homeostasis. Similarly, the primary function of the somatosensory branch of the peripheral nervous system is to collect and interpret sensory information about the environment, allowing the organism to react to or avoid situations that could otherwise have deleterious effects. Consequently, a teleological argument can be made that it is of advantage for the two systems to cooperate and form an “integrated defense system” that benefits from the unique strengths of both subsystems. Indeed, nociceptors, sensory neurons that detect noxious stimuli and elicit the sensation of pain or itch, exhibit potent immunomodulatory capabilities. Depending on the context and the cellular identity of their communication partners, nociceptors can play both pro- or anti-inflammatory roles, promote tissue repair or aggravate inflammatory damage, improve resistance to pathogens or impair their clearance. In light of such variability, it is not surprising that the full extent of interactions between nociceptors and the immune system remains to be established. Nonetheless, the field of peripheral neuroimmunology is advancing at a rapid pace, and general rules that appear to govern the outcomes of such neuroimmune interactions are beginning to emerge. Thus, in this review, we summarize our current understanding of the interaction between nociceptors and, specifically, the myeloid cells of the innate immune system, while pointing out some of the outstanding questions and unresolved controversies in the field. We focus on such interactions within the densely innervated barrier tissues, which can serve as points of entry for infectious agents and, where known, highlight the molecular mechanisms underlying these interactions.

## 1 Introduction

Innate immunity is, in some form, present in virtually all multicellular organisms (1, 2). The functions of the innate immune system include not only elimination of pathogens by direct killing and activation of the adaptive immune system (in organisms in which it is present) (3), but also the induction, modulation and resolution of inflammation, tissue repair (4–6), and control of metabolism (7). Functionally, innate immune cells lack the receptor repertoire diversity of the adaptive immune system, but instead express a selection of germline-encoded receptors that allow recognition of conserved pathogen-associated molecular patterns (PAMPs) as well as damage-associated molecular patterns (DAMPs). Such receptors imbue the innate immune cells with the ability to rapidly respond to the presence of pathogens as well as signs of cellular distress in their environment (8). Furthermore, tissue-resident innate immune cells are strategically located throughout the body and are concentrated in barrier tissues, such as the skin or mucosal surfaces. Thus, in most cases, innate immune responses are initiated within minutes to hours after insult and constitute the body’s first line of defense (9).

On a single-cell level, innate immune cells represent a heterogenous group of leukocytes, which differ in their function, tissue distribution, migratory properties, life-span, turnover, origin, and plasticity. Historically, the various cell types have been differentiated based on their morphology, physiological functions and phenotypes as well as, more recently, their ontogeny. Most innate immune cells are of myeloid origin, i.e. they arise from a common myeloid progenitor (CMP) in the bone marrow (BM) in adults, and from erythro-myeloid progenitors (EMP) in the yolk sac during development (10). Complex relationships between further developmental stages of various myeloid cell types exist, and our understanding of the plasticity of their respective progenitors remains incomplete. Nonetheless, based on phenotypical similarities, myeloid cells can be broadly divided into three families: the mononuclear phagocytes (dendritic cells, macrophages and monocytes) (11), polymorphonuclear granulocytes (neutrophils, basophils and eosinophils) (12), and mast cells (13). In addition to myeloid cells, innate lymphoid cells (ILCs) originating from the common lymphoid progenitor (CLP) have been a focus of intensive research in recent years (14). Notably, neural control of ILCs has been described and reviewed recently (15). Consequently, this review will specifically focus on the effects that nociceptors have on the myeloid immune cell compartment.

Nociceptors are specialized afferent nerve fibers that respond to noxious stimuli such as physical damage, excessive pressure, irritant chemicals, or extremes of temperature and initiate withdrawal/avoidance behavior or irritant removal (16). Based on the modality of the stimuli that they detect, nociceptors can be unimodal or polymodal, i.e. responding to a single or several types of noxious stimuli, respectively (17). Historically, nociceptors have also been classified by the physiological properties of their axons into myelinated A fibers and non-myelinated C fibers, with the latter being the more prevalent group (16–18). More recently, transcriptomic sequencing techniques have begun to define nociceptor subsets based on their gene expression signatures. Indeed, a recent single-cell RNA sequencing study of neurons in murine dorsal root ganglia (DRGs) (19), has proposed classification of nociceptors in into six broad subsets: three non-peptidergic (NP1-3), two peptidergic (PEP1-2) and one tyrosine hydroxylase (TH)-expressing population. Within this classification, the NP1 subset appears to be involved in inflammatory pain, NP3 in inflammatory itch, and all three NP subsets in pruritus in general. The PEP1 population corresponds to thermo-sensitive neurons, whereas PEP2 represent lightly-myelinated Aδ nociceptors (19), which normally respond to mechanical or thermal stimuli (18). Somewhat confusingly, both peptidergic and non-peptidergic nociceptors within this classification express different patterns of neuropeptides (**Table 1**), several of which are known to modulate myeloid cell functions (see below). Of note, more recent studies have suggested dividing nociceptors into as many as 10 different subsets (20). Additionally, even nociceptors belonging within the same subset have been shown to express specific gene patterns depending on the organs they innervate (21), indicating that additional heterogeneity exists and the classification of nociceptors is anything but straightforward.

**Table 1:** Expression of the main neuropeptides with immunoregulatory potential toward myeloid cells across nociceptor subsets at the steady state, as reported by the (19) single cell RNAseq dataset.

	PEP1	PEP2	NP1	NP2	NP3	TH
CGRPα	++++	++++	+	++++	+	+
CGRPβ	++	+++	+++	++++	–	+++
Adrenomedullin	+	+	–	–	–	–
Intermedin	–	–	–	–	–	+
SP	++++	–	+	+	+	+
VIP	–	–	–	–	–	–
PACAP	+++	–	+	+	–	+

**–** no expression detected, **+** expression in <25% of cells, **++** expression in 25 - 50% of cells, **+++** expression in 50 - 75% of cells, and **++++** expression in > 75% of cells.

Nevertheless, nociceptors share certain molecular features that allow their identification and selective experimental manipulation. For example, the NaV1.8 voltage-gated sodium channel is expressed in approximately 90% of all nociceptors and is often considered a pan-nociceptor marker (22). Consequently, although NaV1.8 is also found in certain low-threshold mechanoreceptors (23), NaV1.8-Cre ‘knock-in’ mice (24) have been widely used to target and manipulate nociceptors by genetic means (25–28). Additionally, prominent transient receptor potential (TRP) cation channels have been identified, which often correlate with the specificity of nociceptors for various noxious stimuli including heat (TRPV1), chemical irritants (TRPA1), cold (TRPM8), and others (29). As a result, genetic, as well as chemical means of targeting TRP channels have been developed, which allow for manipulation of nociceptors of a given specificity (30). Experimental means of targeting TRPV1^+^ nociceptors in particular have been widely used in the field, as exemplified by TRPV1-Cre ‘knock-in’ mouse models (31) and the use of TRPV1 agonists, such as capsaicin and resiniferatoxin (RTX), which can hyper-activate and specifically ablate the TRPV1^+^ nociceptors (32, 33).

**Figure 1:**
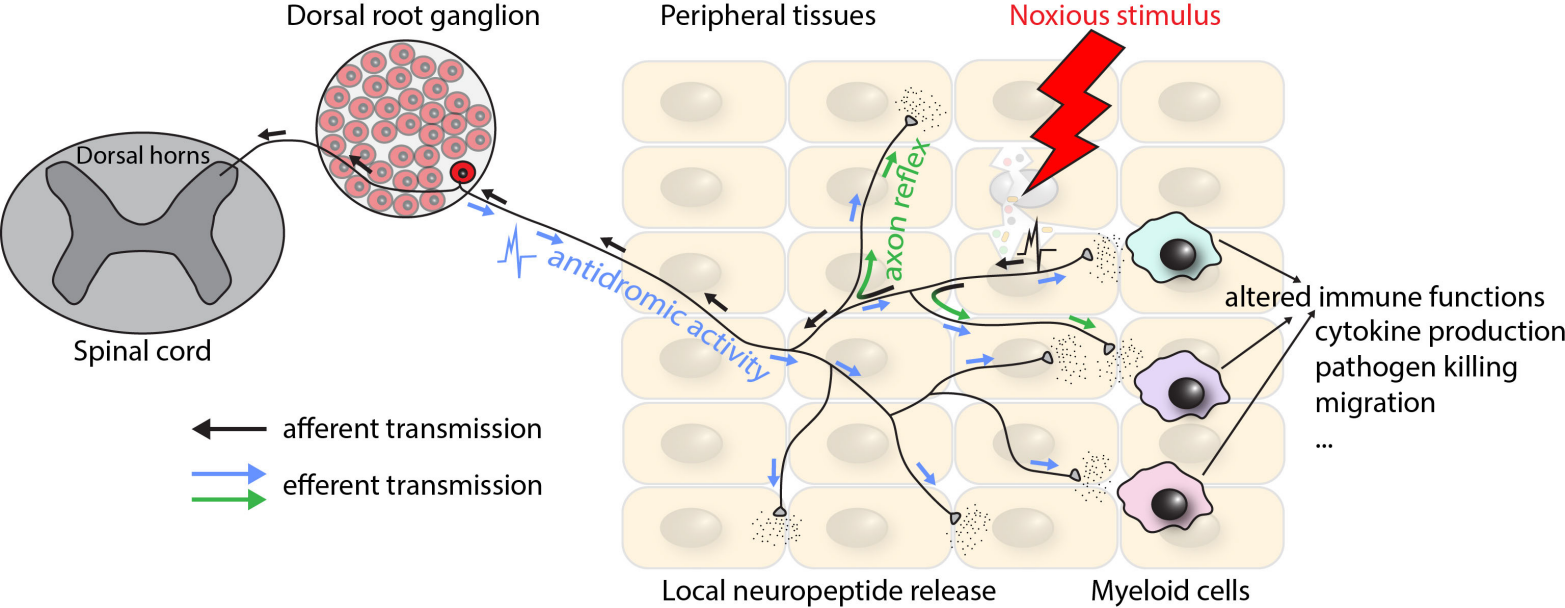
Schematic depiction of the physiological organization of and signal transmission by nociceptors. Arrows indicate the direction of action potential propagation. Black arrows correspond to the afferent transmission of signals elicited by peripheral activation of nociceptors by a noxious stimulus, terminating in the spinal cord and leading to the sensation of pain or itch. Blue and green arrows correspond to the efferent transmission by means of antidromic activity (conduction in the reverse direction) and axon reflex (backpropagation of the action potential through collateral branches) respectively, leading to the peripheral release of neuropeptides.

Anatomically, nociceptor cell bodies are located in the trigeminal ganglia for nociceptors innervating the head and in the DRGs for nociceptors innervating all other parts of the body. Morphologically, nociceptors are pseudo-unipolar neurons with one axon that bifurcates into a proximal and a distal branch. The proximal process terminates in the dorsal horn of the spinal cord or in sensory nuclei of the brainstem, while the distal processes project to peripheral target tissues where they terminate in free endings (18). In addition to their afferent function, nociceptors also exhibit efferent modalities, which are believed to be mediated by several mechanisms including the axon reflex (backpropagation of action potentials through collateral branches) (34) and the antidromic activity (conduction in the reverse direction) (35) (**Figure 1**). Notably, as we will discuss in detail below, efferent functions of nociceptors include the peripheral release of neuropeptides, which act on cells in their proximity, including myeloid leukocytes (34, 35). Of note, the impact of nociceptors and nociceptive neuropeptides on specific target cells depends, at least in part, on the target tissue (36). For example, in the skin, myeloid immune cells are the main targets of nociceptors (26, 37, 38), while in lymph nodes (LNs), the effect on LN-resident myeloid cells is more limited. Instead, non-immune stromal cell types have been identified as the primary communication partners of nociceptors within LNs (21). Lastly, further highlighting how intimately intertwined immune and peripheral nervous systems are, nociceptors express, and are functionally impacted by many of the receptors traditionally thought of as “immune”, including Fc receptors (39), PRRs (40), and checkpoint molecules such as PD-L1 (41).

## 2 Neuropeptides and neuropeptide signaling in immune cells

Neuropeptides are considered the main communication signals that nociceptors emit to impact immune responses, and, indeed, expression of neuropeptide receptors is widespread among myeloid immune cells (**Table 2**). In addition to their immune-modulatory properties, however, neuropeptides also influence numerous other cell types and, as a result, their specific impact on immune cells has often been tested in reductionist systems *in vitro* or *ex vivo*. While this strategy is suitable to identify the molecular mechanisms of action within given cell types, it ignores the tissue context and may not accurately reflect the role that neuropeptides play in physiological settings. Complicating matters further, the concentrations of neuropeptides that can be reached in target tissues due to nociceptor activation remain poorly defined. Consequently, despite a large body of literature detailing the effects of neuropeptides on specific cells in isolation, our understanding of how they impact the function of the immune system as a whole remains far from complete. Finally, it is important to stress that nociceptors are not the only source of neuropeptides (43–45). Consequently, studies in which cells/animals are directly exposed to a neuropeptide (or its inhibitor) can only elucidate effects of the neuropeptide itself, but may not necessarily define the physiological effect of nociceptors.

**Table 2:** Expression of neuropeptide receptors on immune cells based on the RNAseq and microarray data deposited in the Immgen database (42) (https://www.immgen.org/Databrowser19/DatabrowserPage.html).

Neuropeptide	Receptor	DCs	Macrophages	Monocytes	Neutrophils	Eosinophils	Basophils	Mast cells
CGRP/AM/IM	Calcrl	+/++	++/+++	+/++	++	+/++	-/+	+/++/+++
CGRP	RAMP1	++	-/++/+++	++	++/+++	-/+/++	-/++	++/+++
AM/IM	RAMP2	-/+	-/+/++/+++	-/+	-/+	-/+/++	-/+	-/+
	RAMP3	-/+/++	+/++	-/+	-/+	-/+	-/+	+/++
SP	NK1R	-/+	-/+	-/+	-/+	-/+	-/+	-/+
	MRGPRD	-/+	-/+	-/+	-/+	-/++	-/++	++
	MRGPRA1	-/+	-/+	–	–	-/+	-/+	-/+
VIP/PACAP	VPAC1	+	-/+	-/+	+	+	-/+	+/++
	VPAC2	-/+	-/+	-/+	-/+	-/+	-/+	+
PACAP	PAC1	-/+/++	-/+/++	-/+	-/+	-/++	+/++	-/++

**–** no expression, **+** low expression, **++** medium expression, **+++** high expression. Multiple symbols signify uncertainty due to differences between RNAseq and microarray datasets, or variability in expression between cell subsets.

Finally, it should be noted that many neuropeptides, including calcitonin gene-related peptide (CGRP), Substance P (SP) and vasoactive intestinal peptide (VIP), exhibit structural similarities to cationic antimicrobial peptides and, as a result, can exert, at least to some degree, antimicrobial activity (46, 47). The LD_50_ described for most bacterial strains, however, lies in the high micromolar range (48), arguably, well above the concentrations that nociceptor-derived neuropeptides are expected to reach within tissues. Consequently, the physiological relevance of this phenomenon remains unclear, though, a possible role in the regulation of gut microbiota has been suggested (49).

### 2.1 CGRP neuropeptide family

The CGRP family of neuropeptides includes CGRPα (50) and CGRPβ (51), which are thought to be functionally redundant (52), adrenomedullin (AM) (53), and intermedin (54) (also known as adrenomedullin 2 (AM2)). All the CGRP-family neuropeptides signal through a heterodimeric transmembrane receptor comprising a G protein-coupled receptor (GPCR), calcitonin receptor-like receptor (Calcrl), and one of three known receptor-activity modifying proteins (RAMPs) (55). While Calcrl is the signal-transducing, shared subunit, the RAMP proteins dictate the specificity of the complex with RAMP1–Calcrl being the CGRP receptor and RAMP2/3–Calcrl the AM_1/2_ receptors (56). Of the three related neuropeptides, CGRP is the most extensively studied, and its pleiotropic effects are too numerous to be all listed here. The interested reader is referred to an excellent in-depth review (52). Within the scope of the immune system, the effects of CGRP are mostly thought of as anti-inflammatory (25, 28, 57–60), however, several recent studies have highlighted potent pro-inflammatory functions of this neuropeptide in specific contexts (37, 38). The roles of both AM and AM2 remain much less explored; however, they too have been shown to exert some broadly immunoinhibitory functions (61–65).

**Figure 2:**
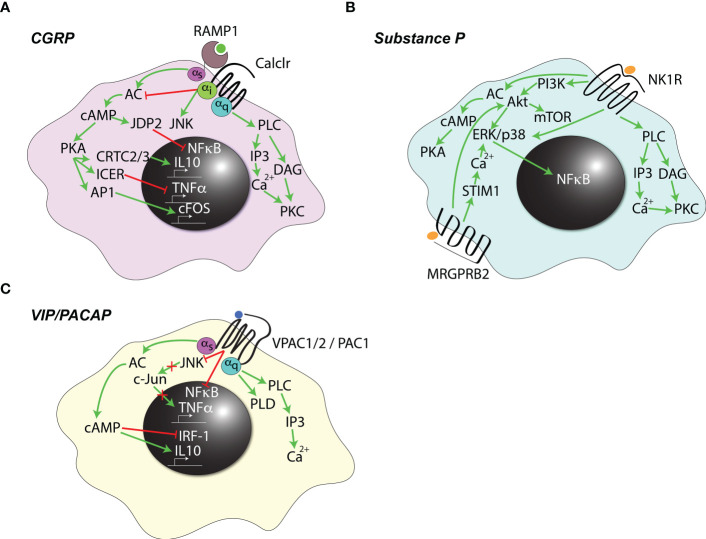
Signaling pathways downstream of neuropeptide receptors. Known signaling pathways initiated by **(A)** calcitonin gene-related peptide (CGRP), **(B)** Substance P, or **(C)** vasoactive intestinal peptide (VIP) and pituitary adenylate cyclase-activating polypeptide (PACAP) signaling in immune cells are summarized. Green arrows indicate activation, red blunt-ended arrows indicate inhibition.

Several types of Gα-proteins are known to couple with the CGRP receptor, leading to the activation of a variety of signaling pathways (**Figure 2A**), some of which appear to be cell-type specific [reviewed in (55)]. Most prominently, Gα_S_ coupling leads to the activation of adenylyl cyclase (AC) (66, 67) and intracellular accumulation of cyclic AMP (cAMP) (68). Conversely, Gα_i_ coupling in certain cell types has been shown to inhibit AC and, instead, to drive JNK activation (69). Finally, CGRP signaling *via* Gα_q_ activates phospholipase C (PLC)-β and protein kinase C (PKC) (70, 71). Consequently, it is tempting to speculate that a preferential engagement of certain Gα subunits by other GPCRs could decrease their overall availability and, thus, regulate the outcomes of CGRP receptor ligation. Lastly, it has been speculated that CGRP may also signal through Gα-independent pathways, however, the biological relevance of this mechanism is unclear (55).

The immuno-inhibitory effects of CGRP are thought to be mainly due to the activation of AC. The resultant accumulation of cAMP activates protein kinase A (PKA) and upregulates the inducible cAMP early repressor (ICER) (72) which, in turn, prevents recruitment of the transcription factor ATF-2 to, among others, the *Tnfa* promoter (73, 74). Additionally, PKA can phosphorylate the cAMP response element-binding protein (CREB), resulting in nuclear translocation of the CREB-regulated transcriptional cofactors (CRTC) 2 and 3, and expression of the anti-inflammatory cytokine IL-10 (75). Finally, cAMP also induces the expression of a transcriptional repressor, Jdp2, which can bind the p65 NF-κB subunit and prevent its docking onto target promoters (57). Additional, cAMP-independent mechanisms are likely also involved, as increased intracellular cAMP concentration alone is not sufficient to mimic the effects of the neuropeptide (76). Inhibition of IκB kinase β phosphorylation and subsequent inhibition of NF-κB signaling was suggested as one such mechanism (77), however, the exact underlying details remain unclear.

Mechanisms of the proinflammatory actions of CGRP in myeloid cells are unknown. In other cell types, however, they, counterintuitively, also appear to rely primarily on the activation of PKA (78, 79). The reasons why the cAMP–PKA axis could act as both pro- and anti-inflammatory are similarly poorly understood, but could be a result of a differential balance between other signaling pathways that exhibit distinct activities in different cell types and/or states.

### 2.2 Substance P

Substance P (SP) is a neuropeptide of the tachykinin family (80) and mainly exerts its functions through one of three GPCRs: the broadly expressed high-affinity neurokinin 1 receptor (NK1R) (81), and the more recently discovered MRGPRB2 and MRGPRA1 which are selectively expressed by mast cells (82–84) and DCs, respectively (85). The actions of SP in most contexts are pro-inflammatory, and several signaling pathways have been implicated (**Figure 2B**) (86, 87). Specifically, NK1R ligation leads to the activation of PLC, which generates the second messengers inositol trisphosphate (IP_3_) and diacyl-glycerol (DAG). These, in turn, can mobilize calcium from intracellular stores and activate PKC (87). In addition, the phosphoinositide 3-kinase (PI3K)-Akt pathway (88) as well as direct activation of the p38 and ERK1/2 mitogen-activated protein kinases (MAPKs) exert proinflammatory effects by triggering NF-κB (89, 90). NK1R can also activate AC with resultant cAMP accumulation and PKA activation (91). Considerably less is known about the signaling pathway downstream of MRGPRB2. Its engagement ultimately leads to a sustained increase in intracellular calcium levels (92) through store-operated calcium entry (SOCE) by the calcium sensor stromal interaction molecule 1 (STIM1) and activation of p38 and ERK MAPKs (93, 94). Additionally, SOCE-independent Akt activation has been reported (94). The mechanistic underpinnings of MRGPRA1 signaling in immune cells remain unknown (85).

### 2.3 VIP and PACAP

Vasoactive intestinal peptide (VIP) and pituitary adenylate cyclase-activating polypeptide (PACAP) are related neuropeptides most commonly upregulated by neurons, including nociceptors, following peripheral nerve injury (95, 96). In nociceptors at steady state, PACAP and VIP often colocalize with CGRP and SP (97, 98), and the tissue content of PACAP is decreased following capsaicin-induced nociceptor depletion (97, 99). The actions of VIP and PACAP are considered to be broadly anti-inflammatory, as these neuropeptides inhibit the release of pro-inflammatory cytokines including TNFα, IL-6, IL-1α and IL-1β, and enhance expression of the anti-inflammatory cytokine IL-10 by several myeloid cell types (**Figure 2C**) (95, 96). These effects are thought to be, at least in part, due to the inhibition of TNFα gene expression, which VIP/PACAP control by two independent mechanisms: blocking NF-κB binding to the *Tnfa* promoter elements, and inhibiting JNK activity, resulting in a decreased phosphorylation of the c-Jun protein and its absence from the CREB complexes docking onto the cAMP response element (CRE) promoter of the *Tnfa* gene (100). Such mechanisms of action are similar to those of CGRP, as described above (see **Figures 2A,C**), making it likely that, in addition to *Tnfa*, they apply also to other pro-inflammatory genes. Additionally, cAMP-dependent inhibition of interferon regulatory factor-1 (IRF-1) transactivation (101) and upregulation of IL-10 have been described (102).

Several receptors for VIP and PACAP have been identified. One receptor, PAC1, is specific for PACAP, whereas VPAC1 and 2 bind indiscriminately to both VIP and PACAP (95). All three receptors participate in immune regulation. In particular, PAC1 and VPAC1 are expressed constitutively on myeloid as well as lymphoid cells, while VPAC2 appears to be inducible, especially in T-cells (103). Like other neuropeptide receptors, VPAC1/2 and PAC1 belong to the GPCR superfamily, and their ligation by agonists leads to coupling to Gα_S_ and activation of AC with downstream accumulation of cAMP (104). Additionally, all three receptors can couple to Gα_q_, and VPAC1/2 can also signal *via* Gα_i_ to activate PLC and calcium mobilization (105). Finally, all three receptors can activate phospholipase D (PLD) (106), however, the underlying mechanism and the relevance of PLD activation for the effects of VIP and PACAP remain poorly understood (105).

## 3 Control of myeloid cells by nociceptors

Nociceptors employ multiple neuropeptide-dependent and -independent means to exert control over myeloid immune cells, whereby the downstream consequences often vary with the target cell type. In the following, we will discuss the most prominent mechanisms by which nociceptors act on each of the major myeloid target cell subsets.

### 3.1 Dendritic cells

Dendritic cells (DCs) are a group of myeloid cells derived from the common DC progenitor (CDP) that include the classical DCs (cDCs) and plasmacytoid DCs (pDCs) (107). pDCs are known for their ability to produce copious amounts of type I and III interferons (IFNs) in response to viral infections, and only exhibit a limited ability to present antigens (108). Notably, pDCs have not been reported to be under the control of nociceptors or respond to nociceptive neuropeptides and will not be further discussed here. Conversely, cDCs (henceforth referred to as DCs) are best known for their ability to take up and present antigens to naive T-cells to activate the adaptive arm of the immune system (109) as well as to induce and maintain tolerance to self and innocuous non-self antigens (110, 111). Additionally, DCs play important roles as sentinel cells within tissues and are involved in pathogen surveillance as well as orchestration of local immune responses by secretion of cytokines, chemokines and other mediators (112). Two subsets of DCs exist – cDC1 and cDC2 – which differ in their phenotypic as well as functional properties. Specifically, cDC1s, identified by their expression of XCR-1 and DNGR-1 (a.k.a. Clec9a), exhibit better ability to present exogenous antigens to CD8^+^ T-lymphocytes, owing to their superior ability to cross-present (i.e. to process and present antigenic peptides from exogenous proteins on MHC class-I complexes). Conversely, cDC2s, identified by their expression of SIRPα and/or CD11b, are generally thought to be superior in their ability to present antigens to CD4^+^ T-lymphocytes, and they comprise the majority of DCs in most tissues (109, 111). Additionally, significant heterogeneity exists among DCs that reside in different anatomic locations (113). This underappreciated diversity could potentially explain some of the seemingly inconsistent observations discussed below.

**Figure 3:**
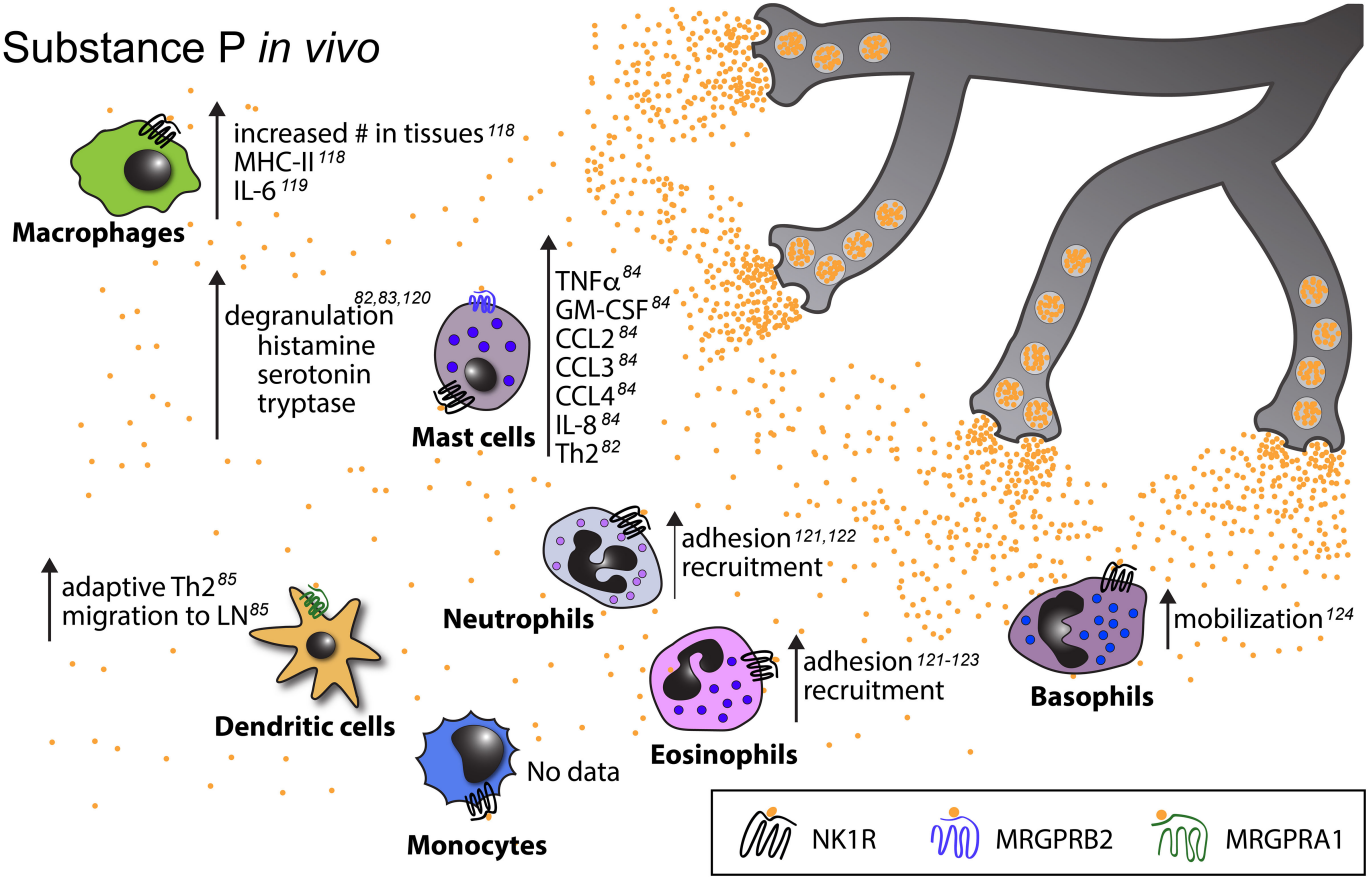
Known *in vivo* effects of substance P on myeloid cells. Upward pointing arrows signify upregulation/activation, downward facing arrows signify downregulation/inhibition.

**Figure 4:**
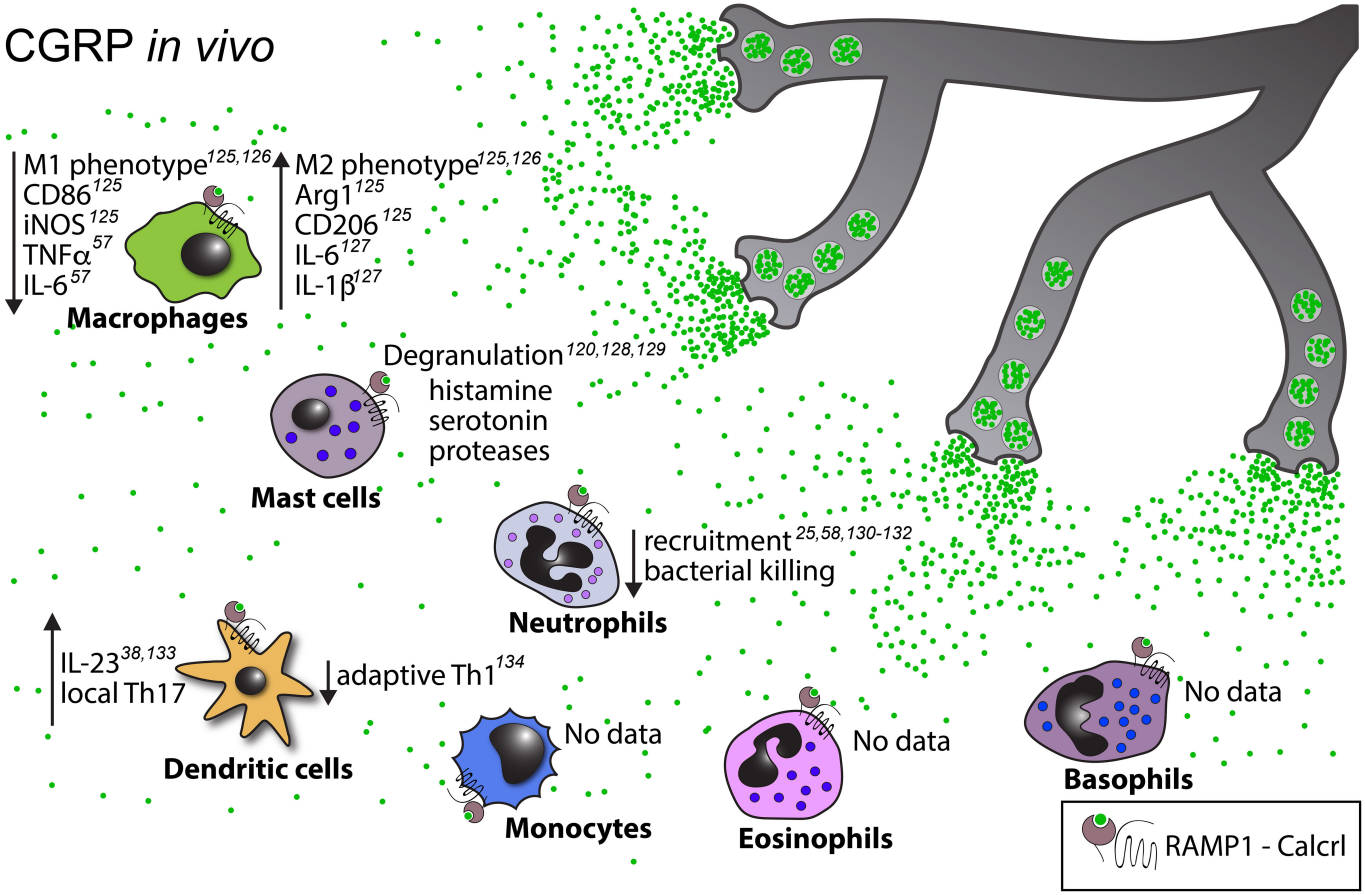
Known *in vivo* effects of CGRP on myeloid cells. Upward pointing arrows signify upregulation/activation, downward facing arrows signify downregulation/inhibition.

**Figure 5:**
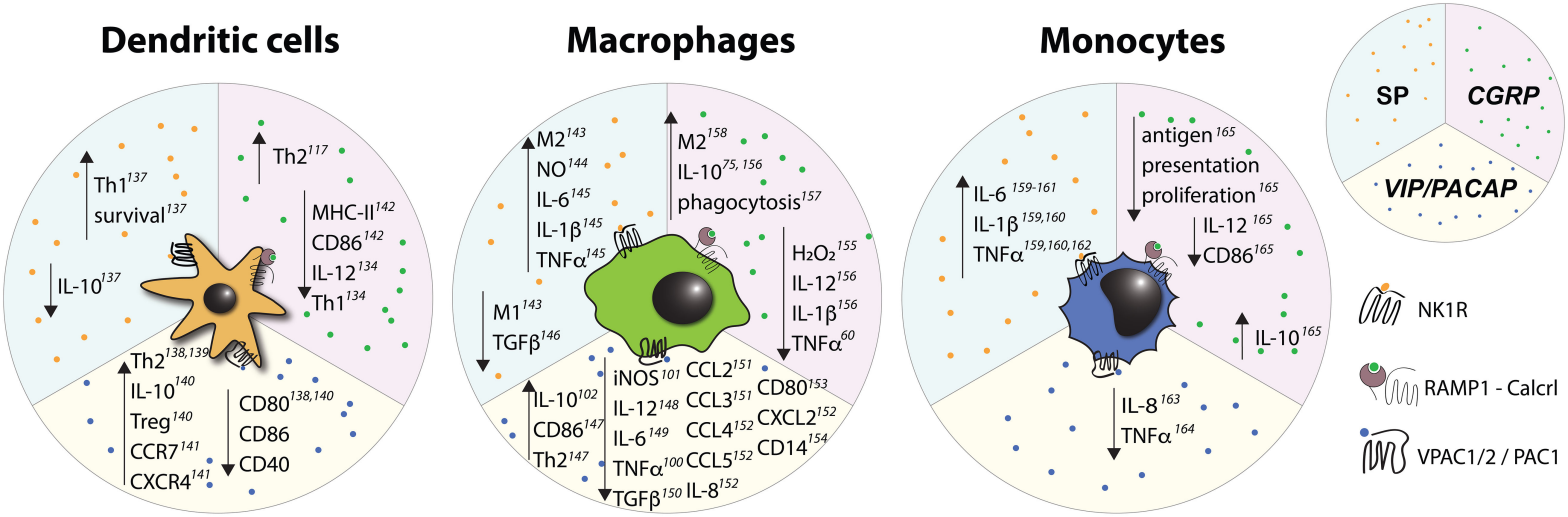
Effects of select nociceptive neuropeptides on mononuclear phagocytes observed *in vitro*. Upward pointing arrows signify upregulation/activation, downward facing arrows signify downregulation/inhibition.

Importantly, both DCs and nociceptors are abundant in peripheral barrier tissues such as the skin and mucosal surfaces, which places them within close proximity of each other (114). For example, DCs and nociceptors engage in direct physical interactions in the murine skin (26, 85). Indeed, DCs in both the skin (26, 38, 85) and the airways (115) are important targets of nociceptor derived communication signals. Similarly, Langerhans cells (LCs) – a subset of specialized dermal phagocytes that are of macrophage lineage but display many functional properties of DCs (116) – are also known to associate with nociceptors, and respond to neuropeptides (117) (**Figures 3**–**5**). DCs are also abundant in secondary lymphoid tissues, including lymph nodes, which are innervated by a unique subset of nociceptors located in the outermost capsular and subcapsular regions (21). However, most lymph node resident DCs are concentrated in the paracortical T cell zone, which is largely devoid of nociceptors, suggesting that lymph node DCs may be relatively less impacted by nociceptors than their counterparts in peripheral barrier tissues.

CGRP effects on DCs: Nociceptor-derived CGRP exerts critical effects on dermal DCs in some, but not all, settings of experimental skin inflammation: One study demonstrated that nociceptor-derived CGRP induced IL-23 production by CD301b^+^ cDC2s upon cutaneous *Candida albicans* infection (38). By contrast, CGRP was dispensable for nociceptor-induced IL-12 and IL-23 production by dermal DCs upon topical treatment with a TLR-7 agonist, Imiquimod (IMQ), in a model of psoriasiform skin inflammation (26). Both studies utilized similar methods to show that nociceptor ablation diminished the cytokine response of dermal DCs. However, in the context of the *C. albicans* infection, local injection of CGRP was sufficient to drive the IL-23 accumulation even in nociceptor-depleted mice, and application of a CGRP antagonist reduced the levels of IL-23 in animals with intact nociceptors (38). Conversely, in the IMQ model, CGRP antagonists had no effect (26). The reasons underlying this discrepancy remain unexplained but could, conceivably, be a result of different subsets of nociceptors responding to a cutaneous fungal infection compared to a defined TLR agonist, or different modalities of activation elicited by the two stimuli in DCs and/or nociceptors. Alternatively, microbiota colonizing the skin could potentially have differentially modulated neuroimmune interactions, as has been recently reported in the gut (118). Lastly, it is pertinent that exposure of mice to stress can synergize with IMQ effects and enhance the accumulation of pro-inflammatory cytokines and tissue inflammation. This effect may be mediated, at least in part, by an increase in the expression of SP (119), however the exact molecular underpinnings have not been identified. Nonetheless, these observations underscore the complexity and context-dependency of neuroimmune interactions. Regardless of the initiation events, in both the IMQ and *C. albicans* models, DC-derived IL-23 activated skin-resident γδT-cells and led to an enhanced local T_H_17 response and increased neutrophil influx (26, 38). Similarly, in the KC-Tie2 model, in which psoriasiform skin inflammation is driven by overexpression of the angiopoietin receptor Tie2 in keratinocytes (120), surgical denervation reduced skin pathology as well as DC numbers and IL-23 expression (121). Exogenous administration of CGRP and SP was sufficient to reverse this effect, and pharmacological blockade of CGRP and SP receptors mimicked the effects of denervation (121). Also in a wound healing model, IMQ-activated TRPA1^+^ nociceptors enhanced IL-23 upregulation in DCs, which, in turn, promoted tissue regeneration (122), however, the role of CGRP or other neuropeptides was not investigated. Further support for CGRP having immuno-stimulatory properties comes from a recent study in which pro-inflammatory cytokines were observed to accumulate in murine skin after optogenetic activation of cutaneous nociceptors in a CGRP-dependent manner (37). Similarly, repeated activation of TRPV1^+^ nociceptors by means of a circumneural sciatic nerve implant induced local inflammation and enhanced the inflammatory response to local adjuvant injection (123). Although neither study determined the exact cellular mechanism, it appears likely that CGRP action on DCs was involved.

Several other studies have demonstrated that CGRP can also have anti-inflammatory and immuno-modulatory effects on DCs. For example, CGRP-deficient mice showed an increase in DC infiltration into the skin after UV-B irradiation overexposure, though whether this was due to a direct effect on DCs or involvement of other cells was not established (124). *In vitro*, LCs exposed to CGRP showed a decreased ability to present antigens to T cells (125, 126), as did classical DCs, in which CGRP downregulated MHC-II and CD86 expression, resulting in decreased T-cell proliferation (127). Furthermore, CGRP treatment was found to suppress T_H_1 differentiation in an *in vitro* DC - T cell co-culture model, and RAMP1^-/-^ mice showed an enhanced T_H_1 response in a model of delayed-type hypersensitivity (DTH) (128). Additionally, CGRP-pretreatment of antigen-pulsed bone marrow-derived DCs (BM-DCs) *in vitro* prior to transfer into naïve mice alleviated the allergic airway inflammation and expansion of allergen-specific T-cells after a subsequent antigenic challenge (129). The underlying mechanism remains unclear, however, significantly increased levels of IL-10 were observed in the tissue (129). Similarly, in the context of 2,4,6-trinitrochlorobenzene-induced contact hypersensitivity (CHS), CGRP injected intradermally during the sensitization phase inhibited the migration of Langerin^+^ dermal DCs to lymph nodes by preventing upregulation of the chemokine receptor CCR7 (130). On the other hand, T-helper 2 (T_H_2)-type responses induced by LCs pretreated with CGRP *in vitro* were enhanced (117). Finally, CGRP was reported to have chemo-attractant properties toward immature but not mature monocyte-derived DCs *in vitro* (131), and to alter the motility of airway mucosal DCs in living lung slices *ex vivo* (132).

In summary, CGRP appears to have the ability to modify functions of DCs in multiple ways, enhancing local immune response within barrier tissues, while downmodulating the DCs’ ability to migrate to lymph nodes and to present antigens to T-cells. Many observations, especially pertaining to the latter, however, have only been made using exogenous administration of CGRP, and it remains unclear whether release of CGRP from nociceptors would be sufficient to induce comparable effects.

AM and intermedin effects on DCs: In contrast to CGRP, much less is known about the effects of AM and intermedin on DCs. However, given the shared signal-transducing element of the CGRP and AM receptors, it is reasonable to speculate that their effects may be largely similar to those of CGRP. Indeed, *in vitro*, AM inhibited LPS-induced maturation of BM-DCs, but it also induced a “semi‐mature tolerogenic” phenotype in unstimulated cells, characterized by intermediate upregulation of CD80, CD86, and indoleamine 2,3‐dioxygenase (IDO) expression (133). Of note, AM-treated BM-DCs were found to express AM themselves (133), however, the relevance of this apparent feed-forward loop remains unclear.

SP effects on DCs: The effects of SP on DCs are generally considered to be pro-inflammatory. *In vitro*, in GM-CSF-induced BM-DCs, SP prevented apoptosis upon withdrawal of GM-CSF through the PI3K-Akt signaling pathway (134), and enhanced T-cell proliferation in a DC – T cell coculture (135). Additionally, SP-treated *in vitro* generated DCs showed decreased IL-10 production and induced an enhanced T_H_1 response when transferred *in vivo* (136). Local administration of a synthetic analog of SP during a gene-gun immunization also resulted in enhanced T_H_1 and cytotoxic T-cell responses (137). Furthermore, pulmonary DCs displayed increased motility when exposed to SP *in vitro* and localized in proximity of SP^+^ nociceptive fibers *in vivo*. Ablation of nociceptors in the lung resulted in a decreased number of DCs and diminished infiltrates after pulmonary antigen challenge (115). Whether this effect was mediated by SP or other nociceptor-derived signals remains to be established. Finally, repeated stress exposure-induced SP accumulation enhanced LC migration out of the skin to the draining lymph-nodes, blocked production of the T_H_2 cytokines IL-4 and IL-5, and enhanced the levels of TNFα and IFNγ, alleviating allergic skin inflammation (138). Nevertheless, there is scant direct evidence for nociceptor-derived SP-mediated control of DC functions under physiological conditions. One recent study reported that the CD301b^+^ subset of skin DCs is activated by TRPV-1^+^ nociceptor-derived SP in an allergen challenge model (85). Such SP-activated DCs showed altered migratory properties and enhanced priming of allergen-specific T_H_2 responses (85). Interestingly, in this study CD301b^+^ DCs recognized SP exclusively through the MRGPRA1 receptor and, in contrast to previous studies (134, 136), the authors were unable to detect expression of any SP receptors on other DC subsets *in vivo* (85). In light of these observations, regulation of the SP receptor expression in DCs emerges as an outstanding question important for our understanding of the interaction between nociceptors and DCs and their outcomes.

VIP and PACAP effects on DCs: The effects of VIP and PACAP on DCs are context-dependent. On one hand, in *in vitro* generated BM-DCs, VIP synergized with TNFα in inducing IL-12 and CD83 expression (139), and VIP/PACAP could also induce CD86 upregulation in immature BM-DCs and allow them to stimulate T-cell proliferation and differentiation into T_H_2 effector cells (140, 141). On the other hand, LPS-activated BM-DCs treated with VIP/PACAP showed impaired upregulation of CD80 and CD86, and decreased ability to stimulate T-cell responses (140). Additionally, BM-DCs that had been differentiated in the presence of VIP/PACAP showed a tolerogenic phenotype, failed to upregulate CD80, CD86, and CD40, expressed high levels of IL-10, and induced the expansion of regulatory T-cells *in vitro* and after transfer *in vivo* (142). Similarly, *in vitro* treatment of antigen-pulsed LCs with VIP prior to their adoptive transfer into previously immunized mice ameliorated LC-dependent DTH responses, possibly due to downregulation of IL-12 and IL-1β, and upregulation of IL-10 (143). Indeed, when administered exogenously *in vivo*, PACAP also suppressed the induction of CHS by modulating Langerhans cell functions (144). However, a more recent study has argued the opposite and showed that denervated mice exhibited an attenuated CHS response, which was improved by repeated intradermal injections of PACAP. Mechanistically, PACAP injections increased the number of dermal DCs that migrated to the draining lymph node and, *in vitro*, the neuropeptide induced upregulation of CCR7 and CXCR4 on immature BM-DCs and improved their ability to migrate toward CCL21 and CXCL12 (145). While it remains unclear whether these *in vitro* and *in vivo* phenomena are connected, the findings suggest that PACAP can control dynamics of DC migration.

Other modes of nociceptor-DC communication: TRPV1^+^ nociceptors have also been shown to be involved in anti-viral responses. Through a yet-to-be defined mechanism, activation of cutaneous nociceptors was sufficient to induce IL-27 expression by dermal CD301b^+^ cells – a heterogenous group of myeloid cells that includes cDC2s as well as monocyte-derived cells – which, in turn, induced expression of anti-viral peptides in keratinocytes. Indeed, skin explants from TRPV-1 deficient mice were more susceptible to HSV infection than those from WT controls (146). Furthermore, DCs isolated from skin-draining lymph nodes of HSV-infected nociceptor-deficient animals were unable to efficiently prime cognate T-cells. Importantly, addition of exogenous antigen rescued the phenotype, indicating that there was no inherent defect in the ability of DCs from nociceptor-depleted mice to activate T-cells but rather that nociceptors control the ability of DCs to acquire and/or process and present antigens to T cells (147).

In summary, nociceptors and nociceptor-derived neuropeptides have the potential to control the trafficking and functions of DCs in multiple ways and, in several cases, a single neuropeptide can exhibit both pro- and anti-inflammatory properties. Many of these observations, however, were only made *in vitro* or in the context of exogenously administered synthetic neuropeptides. Consequently, the extent to which they are relevant for the interaction between nociceptors and DCs under physiological settings often remains unclear. Nevertheless, a picture is beginning to emerge, which suggests that nociceptors may control DC functions in a context-dependent manner through a controlled release of distinct neuropeptides. In particular, the recent *in vivo* data argue in favor of a model in which nociceptors, depending on the type of stimulus encountered, can skew immune responses toward local T_H_17-like (26, 37, 38, 122) or adaptive T_H_2 type responses (85). Whether the diverging responses are mediated by different subsets of nociceptors or the same subset that can itself respond differently to distinct stimuli is currently unclear.

Finally, we note that DCs engage in physical interactions with nociceptors (26, 85), yet the effects of nociceptors that have been described to date are, almost universally, attributed to soluble neuropeptides. Whether there is a role for the physical association of the two cell types *per se* beyond ensuring that DCs are exposed to high concentrations of the locally released neuropeptides remains to be established.

### 3.2 Macrophages

Macrophages comprise a heterogeneous population of tissue-resident myeloid cells with complex ontogeny. In adults, depending on the tissue, macrophage subsets may have a variety of origins: some are derived from yolk sac progenitors and maintained by self-renewal, while others arise from migratory monocytes or other bone marrow derived hematopoietic precursors (148). The immune functions of macrophages include phagocytosis and degradation of cellular debris and foreign objects as well as cytokine production, wound healing and, to a limited degree, antigen presentation (149). Additionally, macrophages have non-immune roles that contribute to the homeostatic functions of various organs including the brain (150), heart (151), lung (152), and liver (153). Macrophages exhibit significant plasticity and, based on phenotypic and functional criteria, are often categorized as M1 or M2 type cells (154). M1 macrophages are characterized by the expression of the inducible nitric oxide synthase (iNOS) as well as the costimulatory molecules CD80 and CD86, and they exhibit pro-inflammatory properties. Conversely, M2 macrophages express the mannose receptor, CD206, and are mostly associated with anti-inflammatory, tissue repair-promoting functions. While the M1/2 nomenclature is often too simplistic to accurately capture the variability observed in macrophages *in vivo* (155), it is, nevertheless, often used as a convenient shorthand. Similar to DCs, macrophages are known to associate with and be impacted by sensory (**Figures 3** **–** **5**) as well as other types of neurons (156) in a variety of tissues including the gut (157), eye (158), and skin (159).

CGRP effects on macrophages: The effect of nociceptive neuropeptides on macrophages is among the earliest recognized examples of nociceptor-immune cell communication. Indeed, CGRP-mediated inhibition of the ability of macrophages to produce H_2_O_2_ and to act as antigen-presenting cells in response to IFNγ was first reported in the late 1980s (160). Subsequent *in vitro* studies have shown that CGRP decreased the expression of other cytokines, including IL-12 and IL-1β, and upregulated IL-10 (161), LIGHT, and SPHK1 through a CREB-dependent mechanism (75). Moreover, CGRP can regulate macrophage polarization *in vitro* (162, 163), by inhibiting LPS-induced degradation of I-κB and promoting IL-4-induced STAT6 phosphorylation, thereby favoring the acquisition of the M2 phenotype (163). Accordingly, in recent *in vivo* experiments CGRP promoted M2 accumulation in two models of post-operative tissue regeneration (162, 164). Additionally, in a β-glucan osteoinflammation model, CGRP released from NaV1.8^+^ nociceptors inhibited osteoclast-mediated bone resorption and decreased TNFα and IL-6 levels (57). *Ex vivo*, CGRP also decreased TNFα production by peritoneal macrophages after LPS stimulation, and CGRP-treated mice were protected from lethal endotoxemia after systemic LPS injection (60). In contrast to these anti-inflammatory actions and similar to its pleiotropic effects on DCs (discussed above), CGRP can also elicit pro-inflammatory responses in macrophages, at least in some settings. For example, in the context of acute postoperative intestinal inflammation, endogenously released CGRP potentiated the expression of the proinflammatory cytokines IL-6 and IL-1β by peritoneal macrophages (165). CGRP also enhanced phagocytic activity in cultured peritoneal macrophages through a cAMP-dependent mechanism (166) and improved their capacity to kill *Leishmania* parasites (167). Interestingly, LPS-activated RAW264.7 macrophage cells *in vitro* (168), as well as macrophages invading an injured nerve site *in vivo* (169), have been shown to produce CGRP themselves, suggesting a regulatory mechanism to promote tissue repair *via* an auto- or paracrine negative feedback loop.

AM and intermedin effects on macrophages: The effects of AM and intermedin on macrophages are not well understood, however, they appear similar to those of CGRP. AM has been shown to decrease the production of TNFα by macrophages *in vitro* (170) and of IL-6 and IL-8 by fallopian tube macrophages *in vivo* (171). Similarly, intermedin can skew macrophage differentiation in white adipose tissue toward the M2 phenotype (172). Conversely, in the NR838 macrophage cell line, AM enhanced the secretion of IL-1β and IL-6, while also reducing the expression of TNFα (173). Just like CGRP, expression of AM has also been shown in the RAW264.7 macrophage cell line as well as in peritoneal macrophages (174) and in macrophages in atherosclerotic plaques (175).

SP effects on macrophages: SP has long been known to induce an oxidative burst in macrophages (176) and to enhance the production of nitric oxide (NO) (177), TNFα, IL-1β, and IL-6 after LPS stimulation *in vitro* (178). Additionally, SP also decreased the production of TGFβ, a cytokine with anti-inflammatory properties (179). SP has been implicated in the mediation of immunological changes induced by stress. For example, in a murine model of sound stress, the percentage of SP^+^ and CGRP^+^ sensory neurons innervating skin was increased (180) and this was accompanied by an SP-dependent increase in MHC-II^+^ macrophage clusters and neurogenic inflammation (181). Likewise, in a model of cold-water stress, SP released from TRPV-1^+^ nerve fibers accumulated in the peritoneal cavity and enhanced IL-6 production by peritoneal macrophages (182). Conversely, in a model of spinal cord injury, intravenous injections of SP decreased the abundance of M1 and increased the abundance of CD206^+^ M2 macrophages at the site of the injury, resulting in a decreased accumulation of IL-6 and TNFα, and an increase in IL-10 (183). Similarly, *in vitro*, SP prevented IFNγ-induced M1 differentiation and activated the PI3K/Akt/mTOR pathway, which promoted differentiation into M2-like tissue repair-promoting macrophages. After adoptive transfer, SP-induced M2 macrophages migrated to sites of tissue injury and improved functional recovery (184).

VIP and PACAP effects on macrophages: VIP and PACAP have been dubbed “macrophage deactivating factors” owing to their ability to inhibit the expression of iNOS (101), proinflammatory cytokines [TNFα (100), IL-6 (185), IL-12 (186)] and chemokines [CCL2, CCL3 (187), CCL4, CCL5, CXCL2 and CXCL8 (IL-8) (188)], and to increase the production of IL-10 (102) by macrophages *in vitro*. Furthermore, VIP and PACAP induced rapid shedding of CD14 – an LPS-coreceptor – thus dampening macrophage responses to bacterial endotoxin (189). Similar to their effect on DCs, VIP/PACAP can, paradoxically, induce upregulation of CD86 on immature macrophages and allow them to stimulate T_H_2-biased adaptive immune responses (190) even though they inhibit CD80 and CD86 upregulation in activated macrophages (191). *In vivo*, exogenous administration of PACAP decreased inflammatory responses by macrophages in CNS and ocular inflammation models, and promoted neuroprotection (192, 193). Conversely, in rat peritoneal macrophages *in vitro*, PACAP has been reported to enhance phagocytosis and production of superoxide anions (194) as well as their adherence and mobility (195), while VIP inhibited TGFβ production (196). Interestingly, pre-treatment of macrophages with VIP and PACAP increased their resistance to HIV infection in a PKA and PKC-dependent manner (197, 198). Of note, while the *in-vitro* observations of immunosuppressive qualities of VIP and PACAP have largely been recapitulated in *in vivo* models in which exogenous neuropeptides were introduced, whether and to what extent nociceptors can and, indeed, do use these neuropeptides to impact macrophage functions *in vivo* is less clear.

TAFA4 effect on macrophages: Because TAFA4 is mostly expressed within the central nervous system (199), its potential role in the periphery had previously been largely overlooked. Recently, however, a Gα_i_-interacting protein (GINIP)-expressing subset of NaV1.8^+^ neurons was shown to express TAFA4 neuropeptide upon UV-irradiation (200). The nociceptor-derived TAFA4 promoted IL-10 expression by macrophages and, consequently, supported the survival of skin-resident anti-inflammatory TIM4^+^ macrophages, resulting in reduced levels of pro-inflammatory cytokines and improved tissue repair (200).

Other modes of nociceptor-macrophage communication: While neuropeptide-dependent communication pathways have been at the forefront of neuroimmunology research in general, non-neuropeptide-dependent modes of communication between nociceptors and macrophages have been described. One such mechanism is the release of HMGB-1 from activated nociceptors. In models of sciatic nerve injury and arthritis, nociceptor-restricted HMGB-1 ablation resulted in decreased inflammation and ameliorated pathology (201). While the HMGB-1-responsive cells were not identified, involvement of macrophages, which express HMGB-1 receptors (202) and play important roles in the development of arthritis (203) appears likely. TRPV1^+^ nociceptors were also implicated in macrophage accumulation, activation, and ROS production in IMQ-induced psoriasiform skin inflammation. In this model, CGRP and SP were responsible for the nocifensive behavior, which was inhibited by treatment with neuropeptide antagonists, but had no effect on the inflammatory response (204), similar to the observations made for DCs in the same model (26). Additionally, in the context of peripheral nerve injury, DRG neurons have been shown to release exosomes containing miR-21. Following phagocytosis of such vesicles, miR-21 induced a pro-inflammatory phenotype in DRG-resident macrophages, characterized by enhanced expression of iNOS, TNFα, and IL-6, and downregulation of CD206 and Arginase (205). Finally, signaling through Toll-like receptors (TLR) and myeloid differentiation factor 88 (MyD88) licenses nociceptors to secrete the chemokine CCL2, resulting in enhanced macrophage infiltration into the DRG (206). The role of such macrophage infiltration in neuropathic pain and neuroinflammation is an important topic of ongoing investigation and has recently been reviewed elsewhere (207).

In summary, nociceptors can utilize multiple neuropeptide-dependent and -independent means to exert control over macrophages, and, *in vivo*, the macrophage response will likely result from a combination of these signals. It is interesting to note that while DCs and macrophages are developmentally and functionally related and their responses to nociceptive neuropeptides bear certain similarities, there appears to be a stark dichotomy in the type of control that nociceptors exhibit over these two cell types. In particular, currently available *in vivo* data suggests that in the case of DCs, nociceptors impact the type of pro-inflammatory immune response, as discussed in the previous section. This is in contrast to macrophages, where the effect of nociceptors appears to focus on controlling the balance between pro- and anti-inflammatory, tissue repair-promoting phenotypes.

### 3.3 Monocytes

Monocytes are circulating phagocytes derived from the common monocyte precursor (CMoP) that are broadly divided into two distinct subsets – classical (also known as inflammatory) and non-classical (a.k.a. patrolling) monocytes – which differ in their phenotype, function and migratory properties. Under inflammatory conditions, monocytes rapidly migrate into the affected tissues where they can undergo diverse differentiation pathways to acquire functional as well as transcriptional properties of DCs or macrophages. Under steady-state conditions, monocytes only emigrate into tissues in small numbers and either help repopulate local macrophage niches or remain in an undifferentiated state to fulfill homeostatic roles and serve as local monocyte reservoirs (208, 209). Interestingly, CGRP, SP, and VIP all exhibit chemotactic properties toward monocytes *in vitro* (210) indicating that at least some monocytes are equipped to sense sensory neuropeptides. Nevertheless, owing to their migratory nature and paucity in uninflamed tissues, direct effects of nociceptors on monocytes *in vivo* have not been studied and our current understanding is limited to *in vitro* effects of neuropeptides (**Figures 3** **–** **5**). Additionally, DCs and macrophages derived from monocytes are rarely differentiated from their bona fide counterparts during analyses and, consequently, whether the effects that nociceptors have on DCs and macrophages *in vivo* are also applicable to monocyte-derived cells remains to be established.

Neuropeptide effects on monocytes: Similar to macrophages, CGRP exerts anti-inflammatory effect on monocytes, including inhibition of proliferation, antigen presentation, upregulation of CD86 (but not CD80), and secretion of IL-12 p40, while at the same time enhancing production of IL-10 in response to *Staphylococcus aureus in vitro* (211). Similarly, human peripheral blood monocytes treated with VIP showed a reduction in the production of TNFα (212) and IL-8 (213) with no effect observed on IL-10 production (212). By contrast, SP exerts primarily pro-inflammatory effects on monocytes. Indeed, several studies have noted that SP can induce the release of IL-1β, TNFα, and IL-6 from monocytes (214, 215) and TNFα from monocyte-derived macrophages (216). A subsequent study, however, reported that human peripheral blood monocytes are unable to respond to SP alone but rather that the neuropeptide synergized with low doses of LPS and enhanced LPS-induced IL-6 expression (217). Consequently, the authors speculated that undetected low levels of LPS in tissue culture media could have been responsible for the proposed cytokine-inducing effects of SP and that SP, in fact, does not act on unstimulated monocytes (217). The controversy has not been fully resolved to date.

In summary, the *in vitro* effects of neuropeptides on monocytes appear similar to their effects on other cells of the mononuclear phagocyte system; however, the absence of *in vivo* data makes it difficult to assess the pathophysiological relevance of this putative communication pathway. One intriguing observation in that context is the reported chemoattractant activity of neuropeptides toward monocytes, which could suggest the possibility of nociceptors controlling monocyte migration and/or localization in tissues.

### 3.4 Polymorphonuclear granulocytes

Polymorphonuclear granulocytes derive from the shared granulocyte-monocyte progenitor (GMP) in the bone marrow and are characterized by the presence of granules in their cytoplasm, which contain a variety of biologically active molecules released upon cellular activation. Granulocytes are often considered the first line of immune defense due to their rapid recruitment to tissues in response to inflammatory stimuli and their potent anti-microbial functions (12).

#### 3.4.1 Eosinophils

Eosinophils play an important role in the pathology of allergic and parasitic diseases as key effectors of T_H_2-type inflammation (218). They respond to cytokines such as IL-5 and IL-13 by proliferating and releasing a variety of cytokines (IL-13, IL-4, IL-25, TNFα, GM-CSF), leukotrienes (C4, D4), prostaglandins, and the contents of their cytotoxic granules, particularly, enzymatic and nonenzymatic cationic proteins, as well as reactive oxygen species (ROS) (218–220).

Although eosinophils also populate the intestinal (221) and uterine mucosa at homeostasis (219, 222), they have been mostly studied in the context of their recruitment in response to allergens and parasites, especially in the airways and the skin. They localize close to sensory as well as parasympathetic neurons both in animal models of asthma and in biopsied lungs of asthma patients (223). In prurigo nodularis, a chronic skin disease characterized by nodules and intense itch, eosinophils are closely associated with CGRP^+^ nociceptive fibers (224) and in atopic dermatitis patients eosinophils are more closely associated with nerve fibers than in healthy controls (225). Finally, although the identity of the neurons was not determined in this context, close nerve-eosinophil contact was also observed in the colon and Peyer’s patches of rats infected with the parasite *Fasciola hepatica* (226). One way eosinophils are postulated to interact with nociceptors is through the CCR3-Eotaxin 1 (CCL11) chemokine pathway. In addition to Eotaxin-1, VCAM-1 expression was also shown on DRG neurons in the presence of nerve growth factor (NGF) *in vitro* (227), which could support adhesion of eosinophils through VLA-4 (integrin α4β1) (228).

**Figure 6:**
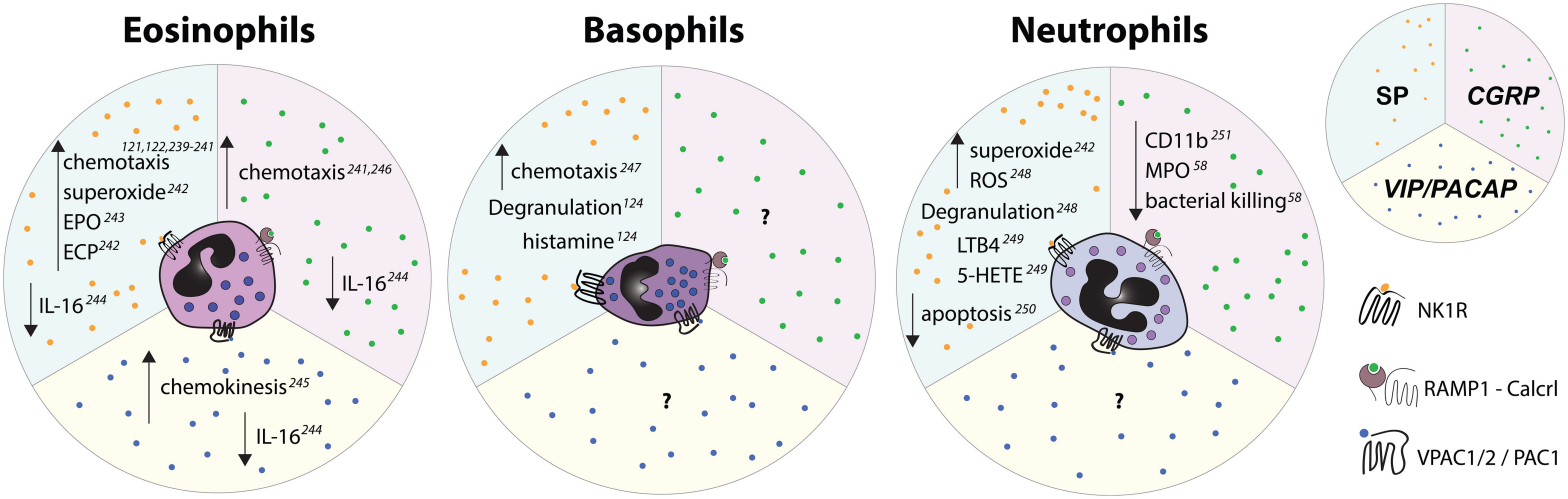
Effects of select nociceptive neuropeptides on polymorphonuclear granulocytes observed *in vitro*. Upward pointing arrows signify upregulation/activation, downward facing arrows signify downregulation/inhibition.

Neuropeptide effects on eosinophils: Like other myeloid leukocytes, eosinophils are also responsive to neuropeptides (**Figures 3**, **4**, **6**) such as CGRP, SP, and VIP, each of which have been shown to decrease IL-16 production by eosinophils *in vitro* (229). When isolated and cultured with SP, Guinea pig eosinophils released eosinophil peroxidase (EPO) (230), a cytotoxic protein contained within “specific” cationic granules (231). The C-terminal fragment of SP also triggers additional eosinophil responses *in vitro*, including the release of another component of the specific granules, the eosinophil cationic protein (ECP) (232), generation of superoxide (232), and inhibition of apoptosis (233). Additionally, equine eosinophils were shown to respond to SP by increased adherence, migration, and superoxide production, although the authors noted that only relatively high SP concentrations were able to elicit these effects (234). SP is also considered an eosinophil chemoattractant (233, 235–237) and intradermal injection of the neuropeptide led to eosinophil recruitment in human volunteers, while CGRP and VIP injections did not (235). Similarly, intranasal SP administration following allergen exposure led to increased eosinophil recruitment in patients with allergic rhinitis (238). Finally, in rat trachea venules, administration of SP increased the numbers of adherent eosinophils in an NK1R-dependent manner (236). However, it is unclear in these experiments whether SP acted directly on eosinophils or rather on endothelial cells to upregulate adhesion molecules [for a review see (239)].

*In vitro* studies with eosinophils from allergic individuals suggest that both CGRP and SP can enhance eosinophil migration toward other chemoattractants, such as leukotriene B4 (237), platelet-activating factor (237, 240), or IL-5 (240). CGRP by itself can induce human eosinophil chemotaxis – directed migration along a concentration gradient, while VIP stimulates chemokinesis – random, directionless motility – by signaling *via* VPAC1 but not VPAC2 (241).

Only a few studies have addressed the effects of nociceptors on eosinophil recruitment and functions *in vivo*: TRPA1-KO mice, which exhibited lower levels of SP, CGRP, and neurokinin A had decreased levels of IL-13, Eotaxin-1, CCL2, RANTES, and IL-5 after an allergic challenge. This phenotype was correlated with a IL-5 decrease in the numbers of eosinophils in the bronchioalveolar lavage fluid (BALF) (242). Similar findings were made in a model of airway inflammation in mice genetically lacking all NaV1.8-expressing nociceptors and mice that were administered QX-314 – a charged derivative of lidocaine and a potent sodium channel blocker, which silences the electrical activity of nociceptors. These mice had fewer eosinophils in their airways and lower levels of IL-5, IL-4, Eotaxin, and TNFα in the BALF (27). Reciprocally, capsaicin treatment, which induces the activation of TRPV1^+^ nociceptors, led to increased eosinophil recruitment (27). Similarly, in a model of allergic asthma, ablation of TRPV1^+^ neurons prevented development of airway hyperreactivity and broncho-constriction phenotypes (243), which are known to be in part mediated by eosinophil major basic protein (MBP) (219, 244). Notably, unlike the observations made in mice lacking NaV1.8^+^ nociceptors (27), no overall changes in the immune cell infiltrate were observed in mice devoid of TRPV1^+^ nociceptors (243). Conceivably, these disparate results might be due to different populations of nociceptors being targeted (NaV1.8^+^ vs TRPV1^+^) or differences in the experimental models themselves. Such differences notwithstanding, a TRPA1 antagonist has shown efficacy in preclinical models of asthma, and has recently entered phase 1 human clinical trials (245), providing an important proof of concept for targeting peripheral neuroimmune interactions in clinical settings.

Taken together, nociceptive neuropeptides have the ability to enhance recruitment and chemotaxis of eosinophils under inflammatory settings and SP in particular can induce eosinophil degranulation and ROS generation. In light of the fact that many neuropeptides can be produced by cells other than nociceptors, the extent to which neuropeptides released specifically by activated nociceptors impact eosinophil functions *in vivo*, however, remains largely unclear.

#### 3.4.2 Basophils

Basophils, which account for ~1% of the blood leukocytes, are recruited to sites of T_H_2-mediated inflammation and are often regarded as the “blood mast cells” (246, 247). It is becoming increasingly clear, however, that basophils have non-redundant functions, especially in their response to haptens and peptide antigens (248). *In vitro*, nociceptor neuropeptides secretoneurin and SP, signaling through NK1R, showed chemoattractant properties comparable to the N-formyl peptide, fMLP, – a known granulocyte chemoattractant – and LPS (249). In mice, intraperitoneal injection of SP led to a marked increase in blood basophil numbers (250). In addition, basophils from patients with chronic spontaneous urticaria had higher expression of NK1R and of SP itself (250). Importantly, the threshold for SP-induced histamine release from such basophils was decreased compared to basophils from healthy subjects (250).

In summary, while it appears that SP enhances the pro-inflammatory functions of basophils (**Figures 3**, **4**, **6**) similarly to what has been described for the other granulocytes (251), our understanding of the neuronal control of their functions is in its infancy.

#### 3.4.3 Neutrophils

Neutrophils are short-lived, sentinel immune cells that comprise 50-70% of all circulating leukocytes in humans and 10-25% in mice (252). As the first responders to pathogen entry, these myeloid cells are equipped with a plethora of effector functions aimed at eliminating pathogens. Specifically, neutrophils phagocytose bacteria, release proteases and oxidants, form neutrophil extracellular traps (NETs), and communicate with other immune cells through cytokine release (253). Importantly, neutrophil activation also has the potential to cause significant collateral damage to the host tissues and, as such, their functions are tightly regulated. Nociceptors have been reported to form close contacts with neutrophils that have emigrated into tissues (28), and nociceptive neuropeptides in particular have been shown to profoundly influence neutrophil migration and functions both *in vitro* and *in vivo* (**Figures 3**, **4**, **6**).

Multiple studies have utilized various depletion or activation techniques to test the impact of nociceptors on neutrophils *in vivo*. These studies usually focus on whether neutrophil recruitment and activity are affected by assessing the expression of adhesion molecules on blood neutrophils, determining neutrophil numbers in the tissue, as well as evaluating the activity of whole tissue myeloperoxidase (MPO), a peroxide-degrading enzyme released during neutrophil degranulation. Although often presented as such, whole tissue MPO activity as a proxy of neutrophil-mediated effector functions has important limitations as it does not account for the production of MPO, albeit at lower levels, by cells other than neutrophils (254). Only a few studies used ex-vivo assays to measure neutrophil-specific MPO and direct antimicrobial properties by co-incubating neutrophils with bacteria in the presence of relevant neuropeptides or nociceptors. Overall, an immunosuppressive effect of nociceptors on neutrophils has been observed. Notably, in rats, electrical stimulation at intensities that activate noxious C fiber afferents led to a reduced accumulation of neutrophils in a model of bradykinin-induced knee joint inflammation (255). While total blood neutrophil numbers remained similar, a marked decrease in L-selectin (CD62L) positive neutrophils in the nociceptor-activated group was reported. Additionally, in a laminar flow assay using blood neutrophils isolated from rats after noxious stimulation, a significant reduction in the number of rolling and tethering cells was observed (255), suggesting that nociceptive fiber stimulation might downmodulate the expression of neutrophil adhesion molecules (256) in parallel with its effects on endothelial cells (239). Building on these original observations and using complementary loss-of-function models, further studies have arrived at similar conclusions: In an LPS-induced subacute airway inflammation model, depletion of TRPV1^+^ nociceptors resulted in a higher lung MPO activity and IL-1β secretion (257). Likewise, in a *Staphylococcus aureus* mouse model of lethal pneumonia, neutrophil recruitment and functions were suppressed by TRPV1^+^ nociceptors, as mice lacking TRPV1^+^ fibers exhibited a higher percentage of neutrophils in the lungs 6h post infection, a lower bacterial burden and better survival (25). Accordingly, an increase in crawling of neutrophils in the subpleural vascular bed was observed using intravital microscopy in the TRPV1^+^ nociceptor-ablated mice (25).

Further studies have also explored the role of nociceptor-neutrophil communication within the gastrointestinal tract in dextran sodium sulfate (DSS) and trinitrobenzene sulfonic acid (TNBS) induced colitis models (258–261). Strikingly, ablation of TRPV1^+^ fibers (258), as well as TRPV1 antagonism (259, 260) resulted in a lower disease score, accompanied by lower tissue MPO activity (258–260). Conversely, however, another study has reported that TRPV1 agonism through daily administration of low doses of capsaicin during DSS colitis in WT rats led to a lower tissue MPO activity (261). While it is possible that such treatment led to the desensitization of the TRPV1^+^ fibers (262), these results indicate that the effects of nociceptors on neutrophils might not be straightforward.

CGRP effects on neutrophils: CGRP acting through the RAMP1-Calcrl receptor is known to have an immunosuppressive effect on neutrophils (25, 263–266). *In vitro*, CGRP prevented LPS-induced upregulation of CD11b on human neutrophils (263) while, *in vivo*, mice lacking RAMP1 exhibited increased infiltration of CD11b^Hi^ neutrophils into the peritoneal cavity and improved antibacterial defense in the early stage of septic peritonitis (265). After myocardial infarction, TRPV1-KO mice showed increased number of recruited neutrophils and higher pro-inflammatory cytokine concentrations (IL-6, TNFα) in the heart, which correlated with lower levels of CGRP and were reversed by exogenous CGRP administration (266). Similarly, in a mouse model of *Streptococcus pyogenes* skin infection, TRPV1^+^ nociceptor-derived CGRP suppressed neutrophil recruitment and functions (58). More recently, analogous observations have also been made in a model of urinary tract infection with uropathogenic *E.coli*, where nociceptor depletion as well as direct CGRP antagonism improved recruitment and functions of neutrophils to the urinary bladder, and resulted in improved bacteria clearance (267). Also *in vitro*, DRG neurons decreased clearance of *S. pyogenes* by bone marrow neutrophils, as did CGRP treatment alone, albeit to a lower degree, suggesting a possible involvement of other nociceptor-derived factors – possibly AM or Intermedin – in suppressing neutrophil antimicrobial activity *in vivo*. Finally, the MPO activity of neutrophils incubated with *S. pyogenes* was also decreased by CGRP in a concentration-dependent manner (58).

SP effects on neutrophils: Consistent with the effects of SP on other myeloid cells, it also amplifies the pro-inflammatory activities of neutrophils. For example, intravenous injection of SP led to increased neutrophil adhesion in rat trachea venules in an NK1R dependent manner (236). Similarly, after intradermal injection of SP in human volunteers, increased numbers of neutrophils in the lumen of dermal venules and the interstitium were observed (235). This phenomenon, however, is likely due to the effects of SP on endothelial cells, which are known to respond to SP by transcriptional upregulation of E-selectin and translocation of P-selectin from Weibel-Palade bodies to the cell surface (235), both adhesion molecules that are critical for the recruitment of blood-borne neutrophils. Nonetheless, direct effects of SP on neutrophils have also been reported, albeit only *in vitro* (232, 268–270). Indeed, SP, through NK1R signaling, enhances neutrophil survival by inhibiting caspase 3-mediated apoptosis (269) and induces superoxide generation (232, 268). Additionally, incubation of human neutrophils with SP resulted in phosphoinositide hydrolysis, an increase in intracellular calcium, activation of NADPH oxidase, and production of reactive oxygen species as well as enhanced exocytosis of azurophilic and specific granules after cytochalasin B treatment (268). Finally, SP has been shown to enhance fMLP-mediated production of arachidonic acid metabolites LTB4 and 5-HETE by human neutrophils, as well as to increase antibody-dependent cellular cytotoxicity (271).

VIP and PACAP effects on neutrophils: The neuropeptides of the VIP/PACAP family seem to have an immunosuppressive effect on neutrophils. In a model of LPS-induced septic shock, VIP/PACAP administration led to lower levels of liver and intestinal MPO activity in a PAC1-dependent manner (272). Furthermore, intratracheal administration of VIP and PACAP analogs prior to IL-1β treatment resulted in a decreased neutrophil infiltrate in the BALF (273), and intraperitoneal administration of recombinant VIP decreased MPO activity in *Aspergillus fumigatus-*infected cornea (274).

In summary, the *in vivo* studies imply that the principal effect of nociceptor activation is attenuation of neutrophil-mediated inflammation, at least in the limited number of experimental systems employed to date. Indeed, neutrophil exposure to several neuropeptides that can be released by nociceptors, particularly CGRP and VIP/PACAP, results in suppression of effector activity. However, at least one nociceptor-derived neuropeptide, SP, can exert potent pro-inflammatory nociceptor-derived activity on neutrophils. While this has only been described *in vitro* and evidence for such effect *in vivo* is currently lacking, it nevertheless hints at an unappreciated degree of complexity in the interaction between nociceptors and neutrophils.

### 3.5 Mast cells

**Figure 7:**
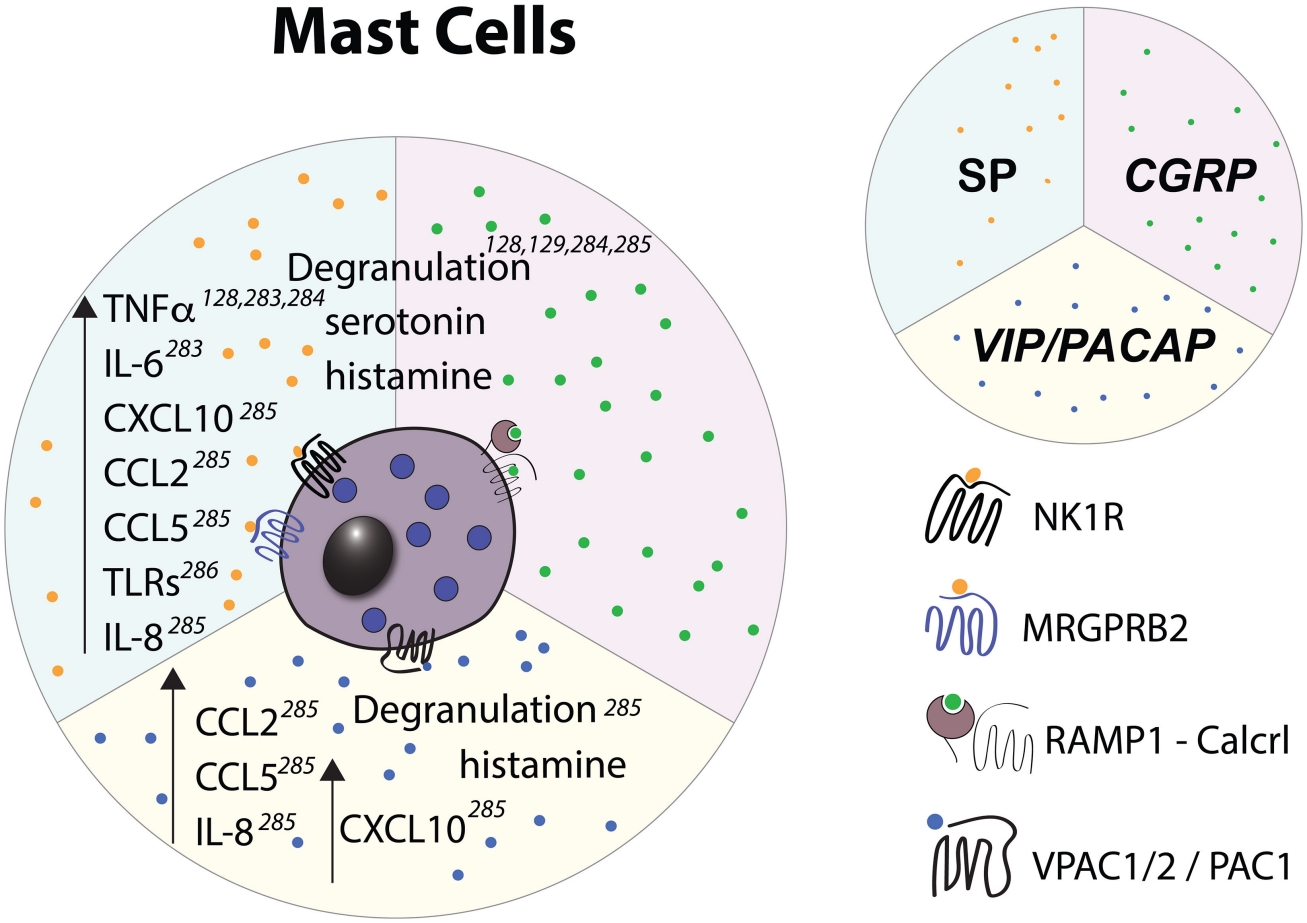
Effects of select nociceptive neuropeptides on mast cells observed *in vitro*. Upward pointing arrows signify upregulation/activation, downward facing arrows signify downregulation/inhibition.

Mast cells (MCs) densely populate barrier tissues and often localize within close proximity of nerve endings (275) and are well known to engage in bi-directional communication with nociceptors (276, 277). MCs play a central role in allergic reactions where, following IgE-mediated crosslinking of Fcϵ receptor I (FcϵRI), they rapidly exocytose storage granules containing mediators such as heparin, histamine, proteases, and cytokines. Granule exocytosis initiates further downstream signaling events that lead to vascular leakage, recruitment of various immune cells, and other components of allergic inflammation (278). Additionally, certain mast cell mediators, in particular histamine and serotonin are potent pruritogens and activate nociceptive fibers that transmit the sensation of itch (279). Neuropeptide-mediated activation of MCs (**Figures 3**, **4**, **7**) is independent of IgE/FcR signaling and results in spatially and temporally distinct patterns of degranulation (280) and release of unique combinations of mediators. Specifically, upon IgE-induced activation, MCs secrete larger and more heterogeneously shaped granules that are able to drain to lymph nodes and influence adaptive immune responses (281) whereas MC activation by neuropeptides induces rapid secretion of small secretory granules that are either not transported to or fail to be retained in draining lymph (280). Additionally, neuropeptide stimulation leads to higher production of diverse pro-inflammatory chemokines, including CCL2 (MCP-1), CCL5 (RANTES), CXCL10 (IP-10), and CXCL8 (IL-8) (282) but limited release of prostaglandin E2 and VEGF (280). These distinct patterns of degranulation and mediator release, potentially along with other, yet to be described molecular features of neuropeptide-mediated MC activation, lead to a more rapid development of vascular leakage but more transient local inflammatory reactions in comparison to anti-IgE-induced activation (280).

MCs in different tissues display marked differences in their phenotype and function, including their neuropeptide receptor expression, granule composition, and cytokine production (283). Mucosal MCs (MMCs) are predominantly found in the gut and contain granules that consist of chondroitin sulfate and either tryptase (in human) or chymase only (in mouse). In contrast, connective tissue MCs (CTMCs) are found in the skin, intestinal submucosa, myocardium (284), nasal epithelium (285), and peritoneum and contain granules that mainly consist of heparin proteoglycans and both tryptase and chymase. Notably, these phenotypic differences have been utilized to generate mouse lines that allow for selective deletion and functional perturbation of either MMCs or CTMCs (286, 287). Although CTMCs and MMCs are ontogenetically distinct (288), they can be induced by the tissue microenvironment to take on phenotypic and functional features of the other subtype (289, 290). Thus, in light of the growing evidence of tissue microenvironment playing a role in regulating MC-nociceptor communication, we will discuss the effect of nociceptor activation on MCs according to their tissue localization:

Nociceptor interactions with CTMCs: SP appears to be the main nociceptive mediator that activates CTMCs, resulting in the release of histamine, proteases, and various chemokines that lead to further innate immune cell recruitment. Although there is some evidence for NK1R expression on human nasal mucosal MCs (291), rat cardiac MCs (292), and dermal MCs within eczematic skin lesions (293), MRGPRB2/X2 appears to be the main receptor for SP in murine and human CTMCs (84). Among the CTMCs, dermal MCs are perhaps the best-characterized population. In the context of tissue damage, SP signals through MRGPRB2/X2 to activate dermal MCs, resulting in their degranulation and release of proinflammatory cytokines (TNFα, GM-CSF) and chemokines (CXCL8, CCL2, 3, and 4) leading to neutrophil recruitment to the site of injury and development of pain hypersensitivity (83). Indeed, injection of staphylococcal enterotoxin B in combination with *Dermatophagoides farinae* extract (house dust mite allergen) activates TRPV1^+^ nociceptors to the release of SP, leading to MC degranulation, cytokine production, influx of eosinophils and neutrophils, and T_H_2-type skin inflammation (82).

Interestingly, unlike activation through the canonical IgE/FcϵRI pathway, SP/MRGPRB2-mediated degranulation results in the release of more tryptase and less histamine and, subsequently, excites the non-histaminergic itch sensory neurons (294). The mechanistic regulation and long-term functional consequence of favoring tryptase release remain to be characterized. Nevertheless, given the ability of tryptase to degrade substrates such as cytokines and neuropeptides (295), it is tempting to speculate that favoring tryptase over histamine could allow for a more controlled pattern of immune activation and rapid return to tissue homeostasis.

In addition to neurogenic inflammation, MRGPRB2/X2-mediated MC activation also facilitates protection against pathogens. In mice lacking functional MRGPRB2, neutrophil recruitment to and clearance of subcutaneously inoculated *S. pyogenes* were impaired (285). Similarly, in a model of *S. aureus* skin infection, MRGPRB2-mediated CTMC degranulation resulted in the release of TNFα, GM-CSF, CXCL8, CCL2, and CCL3 and subsequent recruitment of bacteria-clearing neutrophils and wound healing-promoting CD301b^+^ DCs (296). Instead of SP, however, mastoparan – a different MRGPRB2 agonist – was used to induce CTMC activation. Therefore, whether *S. aureus* infection can physiologically result in the release of SP sufficient to induce CTMC activation in a similar fashion remains to be directly demonstrated. Lastly, in addition to mediating degranulation, SP can also modulate TLR2 expression on MCs (297), which may alter the MC response to subsequent bacterial exposure.

Interestingly, a recent study has shown that a non-peptidergic subset of MrgprD^+^ (Mas-related G-protein-coupled receptor D)-expressing cutaneous sensory afferents modulates gene expression in CTMCs by the release of glutamate (298). Notably, MRGPRB2 was among the downregulated genes, and activation of MrgprD^+^ neurons, which are themselves maintained by skin-resident Langerhans cells, was sufficient to suppress dermal MC responsiveness. Conversely, depletion of the MrgprD^+^ neurons resulted in increased susceptibility to Mrgprb2-mediated irritant dermatitis (298) suggesting that MrgprD^+^ neurons may be responsible for setting an overall tone of CTMC responses in the skin.

In comparison to the skin, less is known about the effects of nociceptive neuropeptides on CTMCs residing in other tissues. *In vitro* studies have demonstrated that SP induces IL-6, TNFα (299) and histamine release from rat peritoneal MCs (300) and histamine from cardiac MCs (292), as well as serotonin and TNFα release from murine peritoneal MCs without a concomitant release of histamine (301, 302). *In vivo*, intraperitoneal inoculation of vancomycin-resistant *E. faecium* into mice with dysfunctional MRGPRB2 resulted in increased bacterial loads compared to controls (285). Based on what has been described for dermal MCs, loss of SP-mediated MC-activation and subsequent defect in the recruitment of bacteria-clearing neutrophils could be the mechanistic underpinning of this phenotype, however, direct experimental evidence is currently lacking. Similarly, murine nasal epithelial MCs also express MRGPRB2, and its dysfunction during nasopharyngeal infection with *S. pneumoniae* resulted in decreased TNFα levels in the nasal lavage fluid (NLF), decreased recruitment of neutrophils and MCs to the nasopharynx, and impaired bacterial clearance compared to control animals (285). Interestingly, in the human nasal mucosa, MCs express NK1R and NK2R but not MRGPRX2 and the functional effect of SP has not been elucidated (291). Overall, more work is necessary to better understand the effect of SP-MRGPRB/X2-mediated CTMC activation within different tissue microenvironments, especially given that SP-MC signaling is implicated in both pathological neurogenic inflammation as well as protection against pathogens.

Molecular mechanisms and functional consequences of CTMC activation by neuropeptides other than SP are poorly understood. CGRP has been shown to induce degranulation and release of serotonin (301) and histamine (303) in peritoneal and dermal MCs and, in a model of contact hypersensitivity (CHS), intradermal injection of CGRP induced release of TNFα from MCs, which resulted in reduced LC density and, consequently, suppressed the CHS phenotype (304). Thus, contrary to other myeloid cells, the effect of CGRP on MCs appears to be preferentially pro- rather than anti-inflammatory. Similar to CGRP, AM was able to induce MC degranulation and histamine release as well as upregulation of VEGF and MCP-1, and increased MC motility *in vitro* (305). However, MC activation by nociceptor-derived AM has not been demonstrated *in vivo.* Interestingly, MCs are a prominent component of solid tumors and, in the tumor microenvironment, can be readily activated by tumor cell-derived AM, resulting in IL-17A release and enhanced tumor growth (306). Lastly, VIP has been shown to induce degranulation and histamine release in rat peritoneal MCs (300) but, paradoxically, suppresses stress-mediated MC degranulation in the testes (307).

Nociceptor interactions with MMCs: Compared to CTMCs, much less is known about neuropeptide-mediated control of MMCs (308). Unlike CTMCs, MMCs do not express MRGPRB/X2 (84) and the expression of NK1R has long been controversial (309). Recent work in human and guinea pigs, however, has demonstrated that NK1R is expressed on intestinal MCs and mediates SP-induced degranulation and release of histamine and protease II (310). In addition to SP, rodent, but not human (309) MMCs also respond to CGRP and VIP by degranulation (311). Interestingly, in murine models of food allergy (312) and intestinal parasitic infection with *Nipppostrongylus brasiliensis* and *Schistosoma mansoni* (313), a significant increase in the density of CGRP^+^ fibers and closely associated MCs was observed in the intestinal mucosa. Similarly, patients with irritable bowel syndrome have an increased number of degranulated MCs localized in close proximity to nerve fibers, and an increase in mucosal tryptase concentrations correlates with the severity and frequency of abdominal pain/discomfort (314). Overall, these observations suggest that the neuropeptide-MC signaling axis plays important, albeit poorly understood roles in the biology of MMCs and their functions in inflammation and protection against pathogens within the intestinal mucosa. Lastly, in a model of SP-induced cystitis in the urinary bladder, MMC-mediated tissue edema and neutrophil infiltration were not dependent on NK1R signaling (315), suggesting that bladder MMCs might express another, yet to be identified SP receptor.

In contrast to other tissues, the lung is uniquely populated by both MMCs and CTMCs, with MMCs being the prevailing type in bronchi, bronchioles, and alveolar parenchyma and CTMCs along the pulmonary vessels and pleura (316). Unlike their murine counterparts (84), human lung MCs do not express MRGPRX2 and, consequently, do not degranulate in response to SP at steady-state (317). However, both the number of MRGPRX2^+^ MCs and overall levels of MRGPRX2 expression were increased in lung biopsies from patients who had died from asthma (318), suggesting that MRGPRX2 may be upregulated in MCs within the inflamed airway microenvironment and potentially play a functional role in airway hyperresponsiveness. Indeed, SP concentration was also elevated in the BAL and sputum of asthmatic patients (319) and SP can induce degranulation of MCs isolated from human BAL (320). Nevertheless, further work is necessary to directly implicate SP-MRGPRB/X2 in the progression of asthma as well as to explore the effect of other neuropeptides on lung MC functions.

Finally, non-barrier, non-mucosal tissues, including the CNS, also contain MCs that are highly responsive to neuropeptides. The dural meninges are densely populated by MCs, which have been reported to form close contacts with nociceptors (321) and respond to SP, VIP, and CGRP by degranulation and histamine release (322). Nevertheless, most *in vivo* work has shown CGRP to be the predominant neuropeptide that mediates nociceptor-MC signaling under pathological conditions, including migraine (323) and post-concussion pain (324). Indeed, epidural infusion of CGRP in rats is sufficient to induce MC degranulation (325). Unlike the rat MCs, however, human meningeal MCs do not appear to express the CGRP receptor (326), and it is unclear whether they can respond to CGRP at all. MCs are also found within specific areas in the underlying brain parenchyma, such as the corpus striatum. Upon systemic VIP administration, these MCs become activated but do not show classic degranulation patterns (327), and their physiological role remains a mystery.

Overall, work thus far has demonstrated important functions of neuropeptide-mediated MC activation in the context of neurogenic inflammation and protection against pathogens in barrier and non-barrier tissues. However, heterogeneity between MC populations present in various tissues remains a confounding factor and a more detailed understanding of how the nociceptor-MC signaling axis functions in tissue-specific immune surveillance is needed. In particular, understanding the function of nociceptor-MC communication in response to pathogens is of outstanding interest, given the MCs’ strategic localization at the host-environment interface, and a growing body of work demonstrating MCs to play critical roles in protection against infection (285, 328).

## 4 Discussion

Although the concept of neuroimmunology in peripheral tissues is not new, progress in our understanding of the operational paradigms that dictate the functional outcomes of such interactions has long been hindered by limitations in the availability of experimental tools and our insufficient knowledge of both the immune and nervous systems *per se*. However, recent progress in multiple areas of scientific inquiry and a convergence of these hitherto separate areas have allowed at least some of these interactions to come into focus. While the field in many ways is still in its infancy, it appears likely that significant headway will be made in years to come.

It is often tempting to over-simplify the effects of nociceptors and neuropeptides on immune cells and categorize them as either pro- or anti-inflammatory. However, we must take into account the interconnectedness and context-dependency that the actions of both the immune and nervous system exhibit. A revealing case in point is the effect of nociceptor-derived CGRP on neutrophil-mediated immune responses. When directly applied, CGRP appears to have potent anti-inflammatory properties, inhibiting neutrophil migration as well as their effector functions. However, through its actions on dendritic cells, CGRP can also enhance the local T_H_17 response, resulting indirectly in an increased production and influx of neutrophils and an enhanced inflammatory response. Teleologically, it is interesting to ponder why the immediate effects of CGRP would prevent neutrophil recruitment if, ultimately, the neuropeptide orchestrates a chain of events that leads to the opposite result. This might be perceived as particularly puzzling given that pain (and concurrent release of neuropeptides) often accompanies barrier breaches, where an increased risk of pathogen invasion should favor the evolution of mechanisms that promote a preemptive recruitment of bacteria-clearing neutrophils. Nevertheless, it is important to take into account the potentially destructive effects that neutrophils can have in inflamed tissues. Indeed, if the threat from a wound is minimal, curtailing of neutrophil accumulation and activation could prevent further tissue damage and favor tissue repair and faster return to homeostasis. On the other hand, larger or infected wounds are likely to cause sustained activation of local immune cells such as the DCs and macrophages alongside the release of CGRP, which together signal a need for a strong immune response, likely overriding the effect that CGRP alone might have on neutrophils. Thus, nociceptors appear to have the ability to fine-tune the immune response in tissues, further enhancing it where needed, while also preventing an excessive activation in situations where it is not.

While it is increasingly clear that nociceptors have the ability to change the parameters of immune responses through the secretion of neuropeptides, the regulation of the processes remains enigmatic. In particular, multiple neuropeptides that often have opposing effects are known to be expressed in the same nociceptive fibers. Consequently, it appears likely that neuropeptide release patterns *in vivo* should be tightly controlled, similarly to what has been described for molecular communication in other branches of the nervous system (329, 330). To that end, it is conceivable that different modalities of activation could result in a spatially and/or temporally distinct pattern of release of distinct neuropeptides from the same fiber. Similar observation have, in fact, been made in rat vagal sensory neuron cultures, in which release of different ratios of CGRP and SP was observed depending on the challenge modality (331). At the same time, at a single-cell/fiber level, nociceptors are known to be non-uniform in their expression of neuropeptides (332, 333). Consequently, it is conceivable that subsets of nociceptors that are distinct in their specificity for various stimuli or their physiological location might inherently show biased patterns of neuropeptide expression to achieve a similar effect. Clearly, further work is needed to establish if and how differential neuropeptide release *in vivo* is controlled and how it impacts immune responses. Additionally, while volume transmission (diffusion-driven effect at a distance) is an accepted mechanism of neuropeptide-dependent communication (334), the scale at which neuropeptide gradients form and the distances at which they can act on various immune cells within tissues remain poorly defined.

It is of note that several feed-forward loops exist in which immune cells exposed to nociceptive neuropeptides are prompted to express the neuropeptides themselves (133, 168, 169, 174, 175). While the functional relevance of such phenomenon has not been investigated, it appears to be an example of a signal-amplifying feed-forward loop. Historically, neuropeptides were thought to be synthesized exclusively in the cell bodies of nociceptors and transported through axons within vesicles to the peripheral nerve endings where they get released (335). More recent studies have, however, demonstrated intra-axonal synthesis of at least some neuropeptides (336), and it remains unclear which of the mechanisms is more important within nociceptors’ axons at the steady state (337). Nonetheless, in either scenario, it appears likely that the amount of neuropeptides readily available in the termini of nociceptors is limited, as the transport along axons has been argued to take hours to days (338), and the scale of local proteosynthesis inside axons is inherently limited by the comparatively small volume of cytoplasm. On the other hand, immune cells have the ability to infiltrate tissues in large quantities, ramp up protein production when activated, and to move within the tissue, perhaps providing a *raison d’être* for such a feed-forward amplifier. Additionally, tissue damage will inevitably result in physical disruption of local nociceptor fibers and hence a possible transient loss of local neuronal neuropeptide sources. It is thus conceivable that myeloid cells present in the affected tissue could provide a substitute source until nociceptor innervation is restored. Experimental evidence in the form of immune cells genetically deficient in the neuropeptides of interest will be necessary to test these ideas.

As discussed throughout this review, to this day, with only a few exceptions, neuropeptide release has widely been considered the main, if not the only, mechanism by which nociceptors exert control over immune cells. Nevertheless, other modalities of interaction could exist, e.g. interactions of surface molecules, direct coupling through gap junctions or tunneling nanotubes, production of secretory vesicles, or release of non-neuropeptide mediators and neurotransmitters. Investigation of such alternative modes of communication could provide worthwhile insights into the nature of the neuroimmune interactions and shed more light on the intricate network of communication in which nociceptors and immune cells engage. Finally, while here we focused specifically on the means by which nociceptors impact on the functions of myeloid immune cells, it is important to stress that the communication between nociceptors and the immune system is bidirectional. Indeed, many of the effects that immune modulators have on nociceptor functions are known and have been reviewed recently (339, 340).

In summary, while a growing body of literature pertaining to the interaction between nociceptors and myeloid cells exists, many outstanding questions remain. In particular, a number of studies investigated the effects of nociceptive neuropeptides on immune cells in isolation or after injecting a neuropeptide into animals, while others have explored the effect of global nociceptor ablation. Although such studies have generated important insights, we are largely lacking a mechanistic understanding of how nociceptor-derived neuropeptides act at the single cell or tissue level. New experimental strategies, such as inducible nociceptor-specific ablation or induction of neuropeptide expression and/or immune cell-specific ablation of neuropeptide receptors could potentially address these questions in the near future. Furthermore, while the interactions between nociceptors and certain cell-types, such as macrophages and mast cells have received much attention, others, such as monocytes or basophils remain largely unexplored.

## Author contributions

PH, M-AM, and YW wrote sections of the manuscript, PH and UHvA generated and edited the final version of the manuscript. All authors contributed to the article and approved the submitted version.

## References

[1] Jones JD Dangl JL. The plant immune system. Nature (2006) 444:323–9. doi: 10.1038/nature05286 17108957

[2] Buchmann K. Evolution of innate immunity: Clues from invertebrates *via* fish to mammals. Front Immunol (2014) 5:459. doi: 10.3389/fimmu.2014.00459 25295041 PMC4172062

[3] Iwasaki A Medzhitov R. Control of adaptive immunity by the innate immune system. Nat Immunol (2015) 16:343–53. doi: 10.1038/ni.3123 PMC4507498 25789684

[4] Watanabe S Alexander M Misharin AV Budinger GRS. The role of macrophages in the resolution of inflammation. J Clin Invest (2019) 129:2619–28. doi: 10.1172/JCI124615 PMC6597225 31107246

[5] Sonnenberg GF Artis D. Innate lymphoid cells in the initiation, regulation and resolution of inflammation. Nat Med (2015) 21:698–708. doi: 10.1038/nm.3892 26121198 PMC4869856

[6] Brazil JC Quiros M Nusrat A Parkos CA. Innate immune cell-epithelial crosstalk during wound repair. J Clin Invest (2019) 129:2983–93. doi: 10.1172/JCI124618 PMC6668695 31329162

[7] Zmora N Bashiardes S Levy M Elinav E. The role of the immune system in metabolic health and disease. Cell Metab (2017) 25:506–21. doi: 10.1016/j.cmet.2017.02.006 28273474

[8] Akira S Uematsu S Takeuchi O. Pathogen recognition and innate immunity. Cell (2006) 124:783–801. doi: 10.1016/j.cell.2006.02.015 16497588

[9] Chaplin DD. Overview of the immune response. J Allergy Clin Immunol (2010) 125:S3–23. doi: 10.1016/j.jaci.2009.12.980 20176265 PMC2923430

[10] De Kleer I Willems F Lambrecht B Goriely S. Ontogeny of myeloid cells. Front Immunol (2014) 5:423. doi: 10.3389/fimmu.2014.00423 25232355 PMC4153297

[11] Chow A Brown BD Merad M. Studying the mononuclear phagocyte system in the molecular age. Nat Rev Immunol (2011) 11:788–98. doi: 10.1038/nri3087 22025056

[12] Geering B Stoeckle C Conus S Simon HU. Living and dying for inflammation: neutrophils, eosinophils, basophils. Trends Immunol (2013) 34:398–409. doi: 10.1016/j.it.2013.04.002 23665135

[13] Krystel-Whittemore M Dileepan KN Wood JG. Mast cell: A multi-functional master cell. Front Immunol (2015) 6:620. doi: 10.3389/fimmu.2015.00620 26779180 PMC4701915

[14] Klose CS Artis D. Innate lymphoid cells as regulators of immunity, inflammation and tissue homeostasis. Nat Immunol (2016) 17:765–74. doi: 10.1038/ni.3489 27328006

[15] Klose CS Artis D. Neuronal regulation of innate lymphoid cells. Curr Opin Immunol (2019) 56:94–9. doi: 10.1016/j.coi.2018.11.002 30530300

[16] Woolf CJ Ma Q. Nociceptors–noxious stimulus detectors. Neuron (2007) 55:353–64. doi: 10.1016/j.neuron.2007.07.016 17678850

[17] Smith ES Lewin GR. Nociceptors: a phylogenetic view. J Comp Physiol A Neuroethol Sens Neural Behav Physiol (2009) 195:1089–106. doi: 10.1007/s00359-009-0482-z PMC2780683 19830434

[18] Dubin AE Patapoutian A. Nociceptors: the sensors of the pain pathway. J Clin Invest (2010) 120:3760–72. doi: 10.1172/JCI42843 PMC2964977 21041958

[19] Usoskin D Furlan A Islam S Abdo H Lonnerberg P Lou D. Unbiased classification of sensory neuron types by large-scale single-cell RNA sequencing. Nat Neurosci (2015) 18:145–53. doi: 10.1038/nn.3881 25420068

[20] Li CL Li KC Wu D Chen Y Luo H Zhao JR. Somatosensory neuron types identified by high-coverage single-cell RNA-sequencing and functional heterogeneity. Cell Res (2016) 26:967. doi: 10.1038/cr.2016.90 27481604 PMC4973338

[21] Huang S Ziegler CGK Austin J Mannoun N Vukovic M Ordovas-Montanes J. Lymph nodes are innervated by a unique population of sensory neurons with immunomodulatory potential. Cell (2021) 184:441–459 e425. doi: 10.1016/j.cell.2020.11.028 33333021 PMC9612289

[22] Abrahamsen B Zhao J Asante CO Cendan CM Marsh S Martinez-Barbera JP. The cell and molecular basis of mechanical, cold, and inflammatory pain. Science (2008) 321:702–5. doi: 10.1126/science.1156916 18669863

[23] Shields SD Ahn HS Yang Y Han C Seal RP Wood JN. Nav1.8 expression is not restricted to nociceptors in mouse peripheral nervous system. Pain (2012) 153:2017–30. doi: 10.1016/j.pain.2012.04.022 22703890

[24] Nassar MA Stirling LC Forlani G Baker MD Matthews EA Dickenson AH. Nociceptor-specific gene deletion reveals a major role for Nav1.7 (PN1) in acute and inflammatory pain. Proc Natl Acad Sci USA (2004) 101:12706–11. doi: 10.1073/pnas.0404915101 PMC515119 15314237

[25] Baral P Umans BD Li L Wallrapp A Bist M Kirschbaum T. Nociceptor sensory neurons suppress neutrophil and gammadelta T cell responses in bacterial lung infections and lethal pneumonia. Nat Med (2018) 24:417–26. doi: 10.1038/nm.4501 PMC6263165 29505031

[26] Riol-Blanco L Ordovas-Montanes J Perro M Naval E Thiriot A Alvarez D. Nociceptive sensory neurons drive interleukin-23-mediated psoriasiform skin inflammation. Nature (2014) 510:157–61. doi: 10.1038/nature13199 PMC4127885 24759321

[27] Talbot S Abdulnour RE Burkett PR Lee S Cronin SJ Pascal MA. Silencing nociceptor neurons reduces allergic airway inflammation. Neuron (2015) 87:341–54. doi: 10.1016/j.neuron.2015.06.007 PMC4506220 26119026

[28] Chiu IM Heesters BA Ghasemlou N Von Hehn CA Zhao F Tran J. Bacteria activate sensory neurons that modulate pain and inflammation. Nature (2013) 501:52–7. doi: 10.1038/nature12479 PMC3773968 23965627

[29] Jardin I Lopez JJ Diez R Sanchez-Collado J Cantonero C Albarran L. TRPs in pain sensation. Front Physiol (2017) 8:392. doi: 10.3389/fphys.2017.00392 28649203 PMC5465271

[30] Moran MM McAlexander MA Biro T Szallasi A. Transient receptor potential channels as therapeutic targets. Nat Rev Drug Discovery (2011) 10:601–20. doi: 10.1038/nrd3456 21804597

[31] Cavanaugh DJ Chesler AT Jackson AC Sigal YM Yamanaka H Grant R. Trpv1 reporter mice reveal highly restricted brain distribution and functional expression in arteriolar smooth muscle cells. J Neurosci (2011) 31:5067–77. doi: 10.1523/JNEUROSCI.6451-10.2011 PMC3087977 21451044

[32] Jeffry JA Yu SQ Sikand P Parihar A Evans MS Premkumar LS. Selective targeting of TRPV1 expressing sensory nerve terminals in the spinal cord for long lasting analgesia. PloS One (2009) 4:e7021. doi: 10.1371/journal.pone.0007021 19753113 PMC2737142

[33] Raisinghani M Pabbidi RM Premkumar LS. Activation of transient receptor potential vanilloid 1 (TRPV1) by resiniferatoxin. J Physiol (2005) 567:771–86. doi: 10.1113/jphysiol.2005.087874 PMC1474234 16037081

[34] Lin Q Wu J Willis WD. Dorsal root reflexes and cutaneous neurogenic inflammation after intradermal injection of capsaicin in rats. J Neurophysiol (1999) 82:2602–11. doi: 10.1152/jn.1999.82.5.2602 10561430

[35] Kress M Guthmann C Averbeck B Reeh PW. Calcitonin gene-related peptide and prostaglandin E2 but not substance P release induced by antidromic nerve stimulation from rat skin *in vitro* . Neuroscience (1999) 89:303–10. doi: 10.1016/s0306-4522(98)00280-2 10051237

[36] Ordovas-Montanes J Rakoff-Nahoum S Huang S Riol-Blanco L Barreiro O von Andrian UH. The regulation of immunological processes by peripheral neurons in homeostasis and disease. Trends Immunol (2015) 36:578–604. doi: 10.1016/j.it.2015.08.007 26431937 PMC4592743

[37] Cohen JA Edwards TN Liu AW Hirai T Jones MR Wu J. Cutaneous TRPV1(+) neurons trigger protective innate type 17 anticipatory immunity. Cell (2019) 178:919–932 e914. doi: 10.1016/j.cell.2019.06.022 31353219 PMC6788801

[38] Kashem SW Riedl MS Yao C Honda CN Vulchanova L Kaplan DH. Nociceptive sensory fibers drive interleukin-23 production from CD301b+ dermal dendritic cells and drive protective cutaneous immunity. Immunity (2015) 43:515–26. doi: 10.1016/j.immuni.2015.08.016 PMC4607048 26377898

[39] Crosson T Wang JC Doyle B Merrison H Balood M Parrin A. FcepsilonR1-expressing nociceptors trigger allergic airway inflammation. J Allergy Clin Immunol (2021) 147:2330–42. doi: 10.1016/j.jaci.2020.12.644 PMC9004488 33453289

[40] Donnelly CR Chen O Ji RR. How do sensory neurons sense danger signals? Trends Neurosci (2020) 43:822–38. doi: 10.1016/j.tins.2020.07.008 PMC7530006 32839001

[41] Meerschaert KA Edwards BS Epouhe AY Jefferson B Friedman R Babyok OL. Neuronally expressed PDL1, not PD1, suppresses acute nociception. Brain Behav Immun (2022) 106:233–46. doi: 10.1016/j.bbi.2022.09.001 PMC10343937 36089217

[42] Heng TS Painter MW Immunological Genome Project Consortium. The immunologicalgenome project: networks of gene expression in immune cells. Nat Immunol (2008) 9:1091–4. doi: 10.1038/ni1008-1091 18800157

[43] Hu R Li YJ Li XH. An overview of non-neural sources of calcitonin gene-related peptide. Curr Med Chem (2016) 23:763–73. doi: 10.2174/0929867323666160210125416 26861004

[44] O’Connor TM O’Connell J O'Brien DI Goode T Bredin CP Shanahan F. The role of substance P in inflammatory disease. J Cell Physiol (2004) 201:167–80. doi: 10.1002/jcp.20061 15334652

[45] Filipsson K Kvist-Reimer M Ahren B. The neuropeptide pituitary adenylate cyclase-activating polypeptide and islet function. Diabetes (2001) 50:1959–69. doi: 10.2337/diabetes.50.9.1959 11522660

[46] El Karim IA Linden GJ Orr DF Lundy FT. Antimicrobial activity of neuropeptides against a range of micro-organisms from skin, oral, respiratory and gastrointestinal tract sites. J Neuroimmunol (2008) 200:11–6. doi: 10.1016/j.jneuroim.2008.05.014 18603306

[47] N'Diaye AR Leclerc C Kentache T Hardouin J Poc CD Konto-Ghiorghi Y. Skin-bacteria communication: Involvement of the neurohormone calcitonin gene related peptide (CGRP) in the regulation of staphylococcus epidermidis virulence. Sci Rep (2016) 6:35379. doi: 10.1038/srep35379 27739485 PMC5064375

[48] Augustyniak D Nowak J Lundy FT. Direct and indirect antimicrobial activities of neuropeptides and their therapeutic potential. Curr Protein Pept Sci (2012) 13:723–38. doi: 10.2174/138920312804871139 PMC3601409 23305360

[49] Aresti Sanz J El Aidy S. Microbiota and gut neuropeptides: a dual action of antimicrobial activity and neuroimmune response. Psychopharmacol (Berl) (2019) 236:1597–609. doi: 10.1007/s00213-019-05224-0 PMC6598950 30997526

[50] Rosenfeld MG Mermod JJ Amara SG Swanson LW Sawchenko PE Rivier J. Production of a novel neuropeptide encoded by the calcitonin gene *via* tissue-specific RNA processing. Nature (1983) 304:129–35. doi: 10.1038/304129a0 6346105

[51] Amara SG Arriza JL Leff SE Swanson LW Evans RM Rosenfeld MG. Expression in brain of a messenger RNA encoding a novel neuropeptide homologous to calcitonin gene-related peptide. Science (1985) 229:1094–7. doi: 10.1126/science.2994212 2994212

[52] Russell FA King R Smillie SJ Kodji X Brain SD. Calcitonin gene-related peptide: physiology and pathophysiology. Physiol Rev (2014) 94:1099–142. doi: 10.1152/physrev.00034.2013 PMC4187032 25287861

[53] Kitamura K Kangawa K Kawamoto M Ichiki Y Nakamura S Matsuo H. Adrenomedullin: a novel hypotensive peptide isolated from human pheochromocytoma. Biochem Biophys Res Commun (1993) 192:553–60. doi: 10.1006/bbrc.1993.1451 8387282

[54] Roh J Chang CL Bhalla A Klein C Hsu SY. Intermedin is a calcitonin/calcitonin gene-related peptide family peptide acting through the calcitonin receptor-like receptor/receptor activity-modifying protein receptor complexes. J Biol Chem (2004) 279:7264–74. doi: 10.1074/jbc.M305332200 14615490

[55] Walker CS Conner AC Poyner DR Hay DL. Regulation of signal transduction by calcitonin gene-related peptide receptors. Trends Pharmacol Sci (2010) 31:476–83. doi: 10.1016/j.tips.2010.06.006 20633935

[56] Hay DL Pioszak AA. Receptor activity-modifying proteins (RAMPs): New insights and roles. Annu Rev Pharmacol Toxicol (2016) 56:469–87. doi: 10.1146/annurev-pharmtox-010715-103120 PMC5559101 26514202

[57] Maruyama K Takayama Y Kondo T Ishibashi KI Sahoo BR Kanemaru H. Nociceptors boost the resolution of fungal osteoinflammation *via* the TRP channel-CGRP-Jdp2 axis. Cell Rep (2017) 19:2730–42. doi: 10.1016/j.celrep.2017.06.002 28658621

[58] Pinho-Ribeiro FA Baddal B Haarsma R O'Seaghdha M Yang NJ Blake KJ. Blocking neuronal signaling to immune cells treats streptococcal invasive infection. Cell (2018) 173:1083–1097 e1022. doi: 10.1016/j.cell.2018.04.006 29754819 PMC5959783

[59] Raud J Lundeberg T Brodda-Jansen G Theodorsson E Hedqvist P. Potent anti-inflammatory action of calcitonin gene-related peptide. Biochem Biophys Res Commun (1991) 180:1429–35. doi: 10.1016/s0006-291x(05)81356-7 1719983

[60] Gomes RN Castro-Faria-Neto HC Bozza PT Soares MB Shoemaker CB David JR. Calcitonin gene-related peptide inhibits local acute inflammation and protects mice against lethal endotoxemia. Shock (2005) 24:590–4. doi: 10.1097/01.shk.0000183395.29014.7c 16317392

[61] Korner C Kuchenbuch T Pfeil U Jung K Padberg W Kummer W. Low-dose adrenomedullin-2/intermedin(8-47) reduces pulmonary ischemia/reperfusion injury. Peptides (2014) 62:49–54. doi: 10.1016/j.peptides.2014.09.022 25290159

[62] Itoh T Obata H Murakami S Hamada K Kangawa K Kimura H. Adrenomedullin ameliorates lipopolysaccharide-induced acute lung injury in rats. Am J Physiol Lung Cell Mol Physiol (2007) 293:L446–452. doi: 10.1152/ajplung.00412.2005 17557801

[63] Xiao F Wang D Kong L Li M Feng Z Shuai B. Intermedin protects against sepsis by concurrently re-establishing the endothelial barrier and alleviating inflammatory response. Nat Commun (2018) 9:2644. doi: 10.1038/s41467-018-05062-2 29980671 PMC6035189

[64] Gonzalez-Rey E Chorny A O'Valle F Delgado M. Adrenomedullin protects from experimental arthritis by down-regulating inflammation and Th1 response and inducing regulatory T cells. Am J Pathol (2007) 170:263–71. doi: 10.2353/ajpath.2007.060596 PMC1762686 17200199

[65] Pedreno M Morell M Robledo G Souza-Moreira L Forte-Lago I Caro M. Adrenomedullin protects from experimental autoimmune encephalomyelitis at multiple levels. Brain Behav Immun (2014) 37:152–63. doi: 10.1016/j.bbi.2013.11.021 PMC3951662 24321213

[66] Edwards RM Stack EJ Trizna W. Calcitonin gene-related peptide stimulates adenylate cyclase and relaxes intracerebral arterioles. J Pharmacol Exp Ther (1991) 257:1020–4. 1710661

[67] Ishikawa T Okamura N Saito A Goto K. Effects of calcitonin gene-related peptide (CGRP) and isoproterenol on the contractility and adenylate cyclase activity in the rat heart. J Mol Cell Cardiol (1987) 19:723–7. doi: 10.1016/s0022-2828(87)80383-8 2826795

[68] Kubota M Moseley JM Butera L Dusting GJ MacDonald PS Martin TJ. Calcitonin gene-related peptide stimulates cyclic AMP formation in rat aortic smooth muscle cells. Biochem Biophys Res Commun (1985) 132:88–94. doi: 10.1016/0006-291x(85)90992-1 2998368

[69] Disa J Parameswaran N Nambi P Aiyar N. Involvement of cAMP-dependent protein kinase and pertussis toxin-sensitive G-proteins in CGRP mediated JNK activation in human neuroblastoma cell line. Neuropeptides (2000) 34:229–33. doi: 10.1054/npep.2000.0810 11021985

[70] Drissi H Lasmoles F Le Mellay V Marie PJ Lieberherr M. Activation of phospholipase C-beta1 *via* Galphaq/11 during calcium mobilization by calcitonin gene-related peptide. J Biol Chem (1998) 273:20168–74. doi: 10.1074/jbc.273.32.20168 9685362

[71] Aiyar N Disa J Stadel JM Lysko PG. Calcitonin gene-related peptide receptor independently stimulates 3',5'-cyclic adenosine monophosphate and Ca2+ signaling pathways. Mol Cell Biochem (1999) 197:179–85. doi: 10.1023/a:1006962221332 10485337

[72] Harzenetter MD Novotny AR Gais P Molina CA Altmayr F Holzmann B. Negative regulation of TLR responses by the neuropeptide CGRP is mediated by the transcriptional repressor ICER. J Immunol (2007) 179:607–15. doi: 10.4049/jimmunol.179.1.607 17579082

[73] Altmayr F Jusek G Holzmann B. The neuropeptide calcitonin gene-related peptide causes repression of tumor necrosis factor-alpha transcription and suppression of ATF-2 promoter recruitment in Toll-like receptor-stimulated dendritic cells. J Biol Chem (2010) 285:3525–31. doi: 10.1074/jbc.M109.066787 PMC2823491 20018859

[74] Kroeger I Erhardt A Abt D Fischer M Biburger M Rau T. The neuropeptide calcitonin gene-related peptide (CGRP) prevents inflammatory liver injury in mice. J Hepatol (2009) 51:342–53. doi: 10.1016/j.jhep.2009.03.022 19464067

[75] Baliu-Pique M Jusek G Holzmann B. Neuroimmunological communication *via* CGRP promotes the development of a regulatory phenotype in TLR4-stimulated macrophages. Eur J Immunol (2014) 44:3708–16. doi: 10.1002/eji.201444553 25316186

[76] Asahina A Moro O Hosoi J Lerner EA Xu S Takashima A. Specific induction of cAMP in Langerhans cells by calcitonin gene-related peptide: relevance to functional effects. Proc Natl Acad Sci USA (1995) 92:8323–7. doi: 10.1073/pnas.92.18.8323 PMC41149 7667288

[77] Ding W Wagner JA Granstein RD. CGRP, PACAP, and VIP modulate Langerhans cell function by inhibiting NF-kappaB activatioN. J Invest Dermatol (2007) 127:2357–67. doi: 10.1038/sj.jid.5700858 17495962

[78] McGillis JP Miller CN Schneider DB Fernandez S Knopf M. Calcitonin gene-related peptide induces AP-1 activity by a PKA and c-fos-dependent mechanism in pre-b cells. J Neuroimmunol (2002) 123:83–90. doi: 10.1016/s0165-5728(01)00484-2 11880153

[79] Kiriyama Y Murayama T Tokumitsu Y Nomura Y. Protein kinase a-dependent IL-6 production induced by calcitonin in human glioblastoma A172 cells. J Neuroimmunol (1997) 76:139–44. doi: 10.1016/s0165-5728(97)00044-1 9184643

[80] Severini C Improta G Falconieri-Erspamer G Salvadori S Erspamer V. The tachykinin peptide family. Pharmacol Rev (2002) 54:285–322. doi: 10.1124/pr.54.2.285 12037144

[81] Fong TM Anderson SA Yu H Huang RR Strader CD. Differential activation of intracellular effector by two isoforms of human neurokinin-1 receptor. Mol Pharmacol (1992) 41:24–30. 1310144

[82] Serhan N Basso L Sibilano R Petitfils C Meixiong J Bonnart C. House dust mites activate nociceptor-mast cell clusters to drive type 2 skin inflammation. Nat Immunol (2019) 20:1435–43. doi: 10.1038/s41590-019-0493-z PMC6858877 31591569

[83] Green DP Limjunyawong N Gour N Pundir P Dong XA. Mast-cell-specific receptor mediates neurogenic inflammation and pain. Neuron (2019) 101:412–420 e413. doi: 10.1016/j.neuron.2019.01.012 30686732 PMC6462816

[84] McNeil BD Pundir P Meeker S Han L Undem BJ Kulka M. Identification of a mast-cell-specific receptor crucial for pseudo-allergic drug reactions. Nature (2015) 519:237–41. doi: 10.1038/nature14022 PMC4359082 25517090

[85] Perner C Flayer CH Zhu X Aderhold PA Dewan ZNA Voisin T. Substance P release by sensory neurons triggers dendritic cell migration and initiates the type-2 immune response to allergens. Immunity (2020) 53:1063–1077 e1067. doi: 10.1016/j.immuni.2020.10.001 33098765 PMC7677179

[86] Mashaghi A Marmalidou A Tehrani M Grace PM Pothoulakis C Dana R. Neuropeptide substance P and the immune response. Cell Mol Life Sci (2016) 73:4249–64. doi: 10.1007/s00018-016-2293-z PMC5056132 27314883

[87] Suvas S. Role of substance P neuropeptide in inflammation, wound healing, and tissue homeostasis. J Immunol (2017) 199:1543–52. doi: 10.4049/jimmunol.1601751 PMC5657331 28827386

[88] Sun J Ramnath RD Tamizhselvi R Bhatia M. Role of protein kinase C and phosphoinositide 3-kinase-Akt in substance P-induced proinflammatory pathways in mouse macrophages. FASEB J (2009) 23:997–1010. doi: 10.1096/fj.08-121756 19029199

[89] Fiebich BL Schleicher S Butcher RD Craig A Lieb K. The neuropeptide substance P activates p38 mitogen-activated protein kinase resulting in IL-6 expression independently from NF-kappa B. J Immunol (2000) 165:5606–11. doi: 10.4049/jimmunol.165.10.5606 11067916

[90] Sun J Ramnath RD Zhi L Tamizhselvi R Bhatia M. Substance P enhances NF-κB transactivation and chemokine response in murine macrophages *via* ERK1/2 and p38 MAPK signaling pathways. Am J Physiology-Cell Physiol (2008) 294:C1586–96. doi: 10.1152/ajpcell.00129.2008 18434625

[91] Duffy MJ Powell D. Stimulation of brain adenylate cyclase activity by the undecapeptide substance P and its modulation by the calcium ion. Biochim Biophys Acta (1975) 385:275–80. doi: 10.1016/0304-4165(75)90355-4 1125264

[92] Subramanian H Gupta K Guo Q Price R Ali H. Mas-related gene X2 (MrgX2) is a novel G protein-coupled receptor for the antimicrobial peptide LL-37 in human mast cells: resistance to receptor phosphorylation, desensitization, and internalization. J Biol Chem (2011) 286:44739–49. doi: 10.1074/jbc.M111.277152 PMC3247983 22069323

[93] Chen X Niyonsaba F Ushio H Nagaoka I Ikeda S Okumura K. Human cathelicidin LL-37 increases vascular permeability in the skin *via* mast cell activation, and phosphorylates MAP kinases p38 and ERK in mast cells. J Dermatol Sci (2006) 43:63–6. doi: 10.1016/j.jdermsci.2006.03.001 16600571

[94] Occhiuto CJ Kammala AK Yang C Nellutla R Garcia M Gomez G. Store-operated calcium entry *via* STIM1 contributes to MRGPRX2 induced mast cell functions. Front Immunol (2019) 10:3143. doi: 10.3389/fimmu.2019.03143 32038646 PMC6985555

[95] Waschek JA. VIP And PACAP: neuropeptide modulators of CNS inflammation, injury, and repair. Br J Pharmacol (2013) 169:512–23. doi: 10.1111/bph.12181 PMC3682700 23517078

[96] Woodley PK Min Q Li Y Mulvey NF Parkinson DB Dun XP. Distinct VIP and PACAP functions in the distal nerve stump during peripheral nerve regeneration. Front Neurosci (2019) 13:1326. doi: 10.3389/fnins.2019.01326 31920495 PMC6920234

[97] Moller K Zhang YZ Hakanson R Luts A Sjolund B Uddman R. Pituitary adenylate cyclase activating peptide is a sensory neuropeptide: immunocytochemical and immunochemical evidence. Neuroscience (1993) 57:725–32. doi: 10.1016/0306-4522(93)90018-b 7508577

[98] Hauser-Kronberger C Hacker GW Albegger K Muss WH Sundler F Arimura A. Distribution of two VIP-related peptides, helospectin and pituitary adenylate cyclase activating peptide (PACAP), in the human upper respiratory system. Regul Pept (1996) 65:203–9. doi: 10.1016/0167-0115(96)00100-0 8897643

[99] Fahrenkrug J Hannibal J. PACAP in visceral afferent nerves supplying the rat digestive and urinary tracts. Ann N Y Acad Sci (1998) 865:542–6. doi: 10.1111/j.1749-6632.1998.tb11233.x 9928066

[100] Delgado M Munoz-Elias EJ Kan Y Gozes I Fridkin M Brenneman DE. Vasoactive intestinal peptide and pituitary adenylate cyclase-activating polypeptide inhibit tumor necrosis factor alpha transcriptional activation by regulating nuclear factor-kB and cAMP response element-binding protein/c-Jun. J Biol Chem (1998) 273:31427–36. doi: 10.1074/jbc.273.47.31427 9813054

[101] Delgado M Munoz-Elias EJ Gomariz RP Ganea D. Vasoactive intestinal peptide and pituitary adenylate cyclase-activating polypeptide prevent inducible nitric oxide synthase transcription in macrophages by inhibiting NF-kappa B and IFN regulatory factor 1 activation. J Immunol (1999) 162:4685–96. doi: 10.4049/jimmunol.162.8.4685 10202009

[102] Delgado M Munoz-Elias EJ Gomariz RP Ganea D. Vasoactive intestinal peptide and pituitary adenylate cyclase-activating polypeptide enhance IL-10 production by murine macrophages: *in vitro* and *in vivo* studies. J Immunol (1999) 162:1707–16. doi: 10.4049/jimmunol.162.3.1707 9973433

[103] Ganea D Hooper KM Kong W. The neuropeptide vasoactive intestinal peptide: direct effects on immune cells and involvement in inflammatory and autoimmune diseases. Acta Physiol (Oxf) (2015) 213:442–52. doi: 10.1111/apha.12427 PMC4484298 25422088

[104] Dickson L Aramori I McCulloch J Sharkey J Finlayson K. A systematic comparison of intracellular cyclic AMP and calcium signalling highlights complexities in human VPAC/PAC receptor pharmacology. Neuropharmacology (2006) 51:1086–98. doi: 10.1016/j.neuropharm.2006.07.017 16930633

[105] Dickson L Finlayson K. VPAC and PAC receptors: From ligands to function. Pharmacol Ther (2009) 121:294–316. doi: 10.1016/j.pharmthera.2008.11.006 19109992

[106] McCulloch DA Lutz EM Johnson MS MacKenzie CJ Mitchell R. Differential activation of phospholipase D by VPAC and PAC1 receptors. Ann N Y Acad Sci (2000) 921:175–85. doi: 10.1111/j.1749-6632.2000.tb06964.x 11193821

[107] Onai N Obata-Onai A Schmid MA Ohteki T Jarrossay D Manz MG. Identification of clonogenic common Flt3+M-CSFR+ plasmacytoid and conventional dendritic cell progenitors in mouse bone marrow. Nat Immunol (2007) 8:1207–16. doi: 10.1038/ni1518 17922016

[108] Reizis B. Plasmacytoid dendritic cells: Development, regulation, and function. Immunity (2019) 50:37–50. doi: 10.1016/j.immuni.2018.12.027 30650380 PMC6342491

[109] Merad M Sathe P Helft J Miller J Mortha A. The dendritic cell lineage: ontogeny and function of dendritic cells and their subsets in the steady state and the inflamed setting. Annu Rev Immunol (2013) 31:563–604. doi: 10.1146/annurev-immunol-020711-074950 23516985 PMC3853342

[110] Ohnmacht C Pullner A King SB Drexler I Meier S Brocker T. Constitutive ablation of dendritic cells breaks self-tolerance of CD4 T cells and results in spontaneous fatal autoimmunity. J Exp Med (2009) 206:549–59. doi: 10.1084/jem.20082394 PMC2699126 19237601

[111] Cabeza-Cabrerizo M Cardoso A Minutti CM Pereira da Costa M Reis e Sousa C. Dendritic cells revisited. Annu Rev Immunol (2021) 39:131–66. doi: 10.1146/annurev-immunol-061020-053707 33481643

[112] Said A Weindl G. Regulation of dendritic cell function in inflammation. J Immunol Res (2015) 2015:743169. doi: 10.1155/2015/743169 26229971 PMC4503598

[113] Sichien D Lambrecht BN Guilliams M Scott CL. Development of conventional dendritic cells: from common bone marrow progenitors to multiple subsets in peripheral tissues. Mucosal Immunol (2017) 10:831–44. doi: 10.1038/mi.2017.8 28198365

[114] Veiga-Fernandes H Mucida D. Neuro-immune interactions at barrier surfaces. Cell (2016) 165:801–11. doi: 10.1016/j.cell.2016.04.041 PMC4871617 27153494

[115] Kradin R MacLean J Duckett S Schneeberger EE Waeber C Pinto C. Pulmonary response to inhaled antigen: neuroimmune interactions promote the recruitment of dendritic cells to the lung and the cellular immune response to inhaled antigen. Am J Pathol (1997) 150:1735–43. PMC1858203 9137097

[116] Doebel T Voisin B Nagao K. Langerhans cells - the macrophage in dendritic cell clothing. Trends Immunol (2017) 38:817–28. doi: 10.1016/j.it.2017.06.008 28720426

[117] Ding W Stohl LL Wagner JA Granstein RD. Calcitonin gene-related peptide biases Langerhans cells toward Th2-type immunity. J Immunol (2008) 181:6020–6. doi: 10.4049/jimmunol.181.9.6020 PMC2679684 18941191

[118] Lai NY Musser MA Pinho-Ribeiro FA Baral P Jacobson A Ma P. Gut-innervating nociceptor neurons regulate peyer's patch microfold cells and SFB levels to mediate Salmonella host defense. Cell (2020) 180:33–49 e22. doi: 10.1016/j.cell.2019.11.014 31813624 PMC6954329

[119] Wang Y Li P Zhang L Fu J Di T Li N. Stress aggravates and prolongs imiquimod-induced psoriasis-like epidermal hyperplasis and IL-1beta/IL-23p40 production. J Leukoc Biol (2020) 108:267–81. doi: 10.1002/JLB.3MA0320-363RR 32421901

[120] Wolfram JA Diaconu D Hatala DA Rastegar J Knutsen DA Lowther A. Keratinocyte but not endothelial cell-specific overexpression of Tie2 leads to the development of psoriasis. Am J Pathol (2009) 174:1443–58. doi: 10.2353/ajpath.2009.080858 PMC2671375 19342373

[121] Ostrowski SM Belkadi A Loyd CM Diaconu D Ward NL. Cutaneous denervation of psoriasiform mouse skin improves acanthosis and inflammation in a sensory neuropeptide-dependent manner. J Invest Dermatol (2011) 131:1530–8. doi: 10.1038/jid.2011.60 PMC3116081 21471984

[122] Wei JJ Kim HS Spencer CA Brennan-Crispi D Zheng Y Johnson NM. Activation of TRPA1 nociceptor promotes systemic adult mammalian skin regeneration. Sci Immunol (2020) 5. doi: 10.1126/sciimmunol.aba5683 PMC7703669 32859683

[123] Michoud F Seehus C Schonle P Brun N Taub D Zhang Z. Epineural optogenetic activation of nociceptors initiates and amplifies inflammation. Nat Biotechnol (2021) 39:179–85. doi: 10.1038/s41587-020-0673-2 PMC7878280 32958958

[124] La Russa F Lopes DM Hobbs C Argunhan F Brain S Bevan S. Disruption of the sensory system affects sterile cutaneous inflammation *in vivo* . J Invest Dermatol (2019) 139:1936–1945 e1933. doi: 10.1016/j.jid.2019.01.037 30974165

[125] Hosoi J Murphy GF Egan CL Lerner EA Grabbe S Asahina A. Regulation of Langerhans cell function by nerves containing calcitonin gene-related peptide. Nature (1993) 363:159–63. doi: 10.1038/363159a0 8483499

[126] Torii H Hosoi J Asahina A Granstein RD. Calcitonin gene-related peptide and Langerhans cell function. J Investig Dermatol Symp Proc (1997) 2:82–6. doi: 10.1038/jidsymp.1997.16 9487021

[127] Carucci JA Ignatius R Wei Y Cypess AM Schaer DA Pope M. Calcitonin gene-related peptide decreases expression of HLA-DR and CD86 by human dendritic cells and dampens dendritic cell-driven T cell-proliferative responses *via* the type I calcitonin gene-related peptide receptor. J Immunol (2000) 164:3494–9. doi: 10.4049/jimmunol.164.7.3494 10725702

[128] Mikami N Sueda K Ogitani Y Otani I Takatsuji M Wada Y. Calcitonin gene-related peptide regulates type IV hypersensitivity through dendritic cell functions. PloS One (2014) 9:e86367. doi: 10.1371/journal.pone.0086367 24466057 PMC3897726

[129] Rochlitzer S Veres TZ Kuhne K Prenzler F Pilzner C Knothe S. The neuropeptide calcitonin gene-related peptide affects allergic airway inflammation by modulating dendritic cell function. Clin Exp Allergy (2011) 41:1609–21. doi: 10.1111/j.1365-2222.2011.03822.x 21752117

[130] Mikami N Matsushita H Kato T Kawasaki R Sawazaki T Kishimoto T. Calcitonin gene-related peptide is an important regulator of cutaneous immunity: effect on dendritic cell and T cell functions. J Immunol (2011) 186:6886–93. doi: 10.4049/jimmunol.1100028 21551361

[131] Dunzendorfer S Kaser A Meierhofer C Tilg H Wiedermann CJ. Cutting edge: peripheral neuropeptides attract immature and arrest mature blood-derived dendritic cells. J Immunol (2001) 166:2167–72. doi: 10.4049/jimmunol.166.4.2167 11160268

[132] Voedisch S Rochlitzer S Veres TZ Spies E Braun A. Neuropeptides control the dynamic behavior of airway mucosal dendritic cells. PloS One (2012) 7:e45951. doi: 10.1371/journal.pone.0045951 23049899 PMC3458805

[133] Rulle S Ah Kioon MD Asensio C Mussard J Ea HK Boissier MC. Adrenomedullin, a neuropeptide with immunoregulatory properties induces semi-mature tolerogenic dendritic cells. Immunology (2012) 136:252–64. doi: 10.1111/j.1365-2567.2012.03577.x PMC3403265 22348691

[134] Janelsins BM Mathers AR Tkacheva OA Erdos G Shufesky WJ Morelli AE. Proinflammatory tachykinins that signal through the neurokinin 1 receptor promote survival of dendritic cells and potent cellular immunity. Blood (2009) 113:3017–26. doi: 10.1182/blood-2008-06-163121 PMC2662645 18987361

[135] Lambrecht BN Germonpre PR Everaert EG Carro-Muino I De Veerman M de Felipe C. Endogenously produced substance P contributes to lymphocyte proliferation induced by dendritic cells and direct TCR ligation. Eur J Immunol (1999) 29:3815–25. doi: 10.1002/(SICI)1521-4141(199912)29:12<3815::AID-IMMU3815>3.0.CO;2-# 10601989

[136] Janelsins BM Sumpter TL Tkacheva OA Rojas-Canales DM Erdos G Mathers AR. Neurokinin-1 receptor agonists bias therapeutic dendritic cells to induce type 1 immunity by licensing host dendritic cells to produce IL-12. Blood (2013) 121:2923–33. doi: 10.1182/blood-2012-07-446054 PMC3624938 23365459

[137] Mathers AR Tckacheva OA Janelsins BM Shufesky WJ Morelli AE Larregina AT. *In vivo* signaling through the neurokinin 1 receptor favors transgene expression by Langerhans cells and promotes the generation of Th1- and Tc1-biased immune responses. J Immunol (2007) 178:7006–17. doi: 10.4049/jimmunol.178.11.7006 17513750

[138] Pavlovic S Liezmann C Blois SM Joachim R Kruse J Romani N. Substance P is a key mediator of stress-induced protection from allergic sensitization *via* modified antigen presentation. J Immunol (2011) 186:848–55. doi: 10.4049/jimmunol.0903878 21172866

[139] Delneste Y Herbault N Galea B Magistrelli G Bazin I Bonnefoy JY. Vasoactive intestinal peptide synergizes with TNF-alpha in inducing human dendritic cell maturation. J Immunol (1999) 163:3071–5. doi: 10.4049/jimmunol.163.6.3071 10477571

[140] Delgado M Reduta A Sharma V Ganea D. VIP/PACAP oppositely affects immature and mature dendritic cell expression of CD80/CD86 and the stimulatory activity for CD4(+) T cells. J Leukoc Biol (2004) 75:1122–30. doi: 10.1189/jlb.1203626 15020654

[141] Delgado M Leceta J Gomariz RP Ganea D. Vasoactive intestinal peptide and pituitary adenylate cyclase-activating polypeptide stimulate the induction of Th2 responses by up-regulating B7.2 expression. J Immunol (1999) 163:3629–35. doi: 10.4049/jimmunol.163.7.3629 10490956

[142] Delgado M Gonzalez-Rey E Ganea D. The neuropeptide vasoactive intestinal peptide generates tolerogenic dendritic cells. J Immunol (2005) 175:7311–24. doi: 10.4049/jimmunol.175.11.7311 16301637

[143] Kodali S Ding W Huang J Seiffert K Wagner JA Granstein RD. Vasoactive intestinal peptide modulates Langerhans cell immune function. J Immunol (2004) 173:6082–8. doi: 10.4049/jimmunol.173.10.6082 15528344

[144] Kodali S Friedman I Ding W Seiffert K Wagner JA Granstein RD. Pituitary adenylate cyclase-activating polypeptide inhibits cutaneous immune function. Eur J Immunol (2003) 33:3070–9. doi: 10.1002/eji.200324085 14579275

[145] Yamamoto Y Otsuka A Ishida Y Wong LS Seidel JA Nonomura Y. Pituitary adenylate cyclase-activating polypeptide promotes cutaneous dendritic cell functions in contact hypersensitivity. J Allergy Clin Immunol (2021) 148:858–66. doi: 10.1016/j.jaci.2021.02.005 33609627

[146] Lei V Handfield C Kwock JT Kirchner SJ Lee MJ Coates M. Skin injury activates a rapid TRPV1-dependent antiviral protein response. J Invest Dermatol (2022) 142:2249–2259 e2249. doi: 10.1016/j.jid.2021.11.041 35007556 PMC9259761

[147] Filtjens J Roger A Quatrini L Wieduwild E Gouilly J Hoeffel G. Nociceptive sensory neurons promote CD8 T cell responses to HSV-1 infection. Nat Commun (2021) 12:2936. doi: 10.1038/s41467-021-22841-6 34006861 PMC8131384

[148] Epelman S Lavine KJ Randolph GJ. Origin and functions of tissue macrophages. Immunity (2014) 41:21–35. doi: 10.1016/j.immuni.2014.06.013 25035951 PMC4470379

[149] Wynn TA Chawla A Pollard JW. Macrophage biology in development, homeostasis and disease. Nature (2013) 496:445–55. doi: 10.1038/nature12034 PMC3725458 23619691

[150] Eroglu C Barres BA. Regulation of synaptic connectivity by glia. Nature (2010) 468:223–31. doi: 10.1038/nature09612 PMC4431554 21068831

[151] Hulsmans M Clauss S Xiao L Aguirre AD King KR Hanley A. Macrophages facilitate electrical conduction in the heart. Cell (2017) 169:510–522 e520. doi: 10.1016/j.cell.2017.03.050 28431249 PMC5474950

[152] Hussell T Bell TJ. Alveolar macrophages: plasticity in a tissue-specific context. Nat Rev Immunol (2014) 14:81–93. doi: 10.1038/nri3600 24445666

[153] Dixon LJ Barnes M Tang H Pritchard MT Nagy LE. Kupffer cells in the liver. Compr Physiol (2013) 3:785–97. doi: 10.1002/cphy.c120026 PMC4748178 23720329

[154] Mills CD Kincaid K Alt JM Heilman MJ Hill AM. M-1/M-2 macrophages and the Th1/Th2 paradigm. J Immunol (2000) 164:6166–73. doi: 10.4049/jimmunol.164.12.6166 10843666

[155] Mosser DM Edwards JP. Exploring the full spectrum of macrophage activation. Nat Rev Immunol (2008) 8:958–69. doi: 10.1038/nri2448 PMC2724991 19029990

[156] Kolter J Kierdorf K Henneke P. Origin and differentiation of nerve-associated macrophages. J Immunol (2020) 204:271–9. doi: 10.4049/jimmunol.1901077 31907269

[157] Gabanyi I Muller PA Feighery L Oliveira TY Costa-Pinto FA Mucida D. Neuro-immune interactions drive tissue programming in intestinal macrophages. Cell (2016) 164:378–91. doi: 10.1016/j.cell.2015.12.023 PMC4733406 26777404

[158] Seyed-Razavi Y Chinnery HR McMenamin PG. A novel association between resident tissue macrophages and nerves in the peripheral stroma of the murine cornea. Invest Ophthalmol Vis Sci (2014) 55:1313–20. doi: 10.1167/iovs.13-12995 24458151

[159] Kolter J Feuerstein R Zeis P Hagemeyer N Paterson N d'Errico P. A subset of skin macrophages contributes to the surveillance and regeneration of local nerves. Immunity (2019) 50:1482–1497 e1487. doi: 10.1016/j.immuni.2019.05.009 31201094

[160] Nong YH Titus RG Ribeiro JM Remold HG. Peptides encoded by the calcitonin gene inhibit macrophage function. J Immunol (1989) 143:45–9. doi: 10.4049/jimmunol.143.1.45 2543703

[161] Torii H Hosoi J Beissert S Xu S Fox FE Asahina A. Regulation of cytokine expression in macrophages and the Langerhans cell-like line XS52 by calcitonin gene-related peptide. J Leukoc Biol (1997) 61:216–23. doi: 10.1002/jlb.61.2.216 9021928

[162] Yuan Y Jiang Y Wang B Guo Y Gong P Xiang L. Deficiency of calcitonin gene-related peptide affects macrophage polarization in osseointegration. Front Physiol (2020) 11:733. doi: 10.3389/fphys.2020.00733 32848807 PMC7412000

[163] Duan JX Zhou Y Zhou AY Guan XX Liu T Yang HH. Calcitonin gene-related peptide exerts anti-inflammatory property through regulating murine macrophages polarization *in vitro* . Mol Immunol (2017) 91:105–13. doi: 10.1016/j.molimm.2017.08.020 28892747

[164] Matsui S Tanaka M Kamiyoshi A Sakurai T Ichikawa-Shindo Y Kawate H. Endogenous calcitonin gene-related peptide deficiency exacerbates postoperative lymphedema by suppressing lymphatic capillary formation and M2 macrophage accumulation. Am J Pathol (2019) 189:2487–502. doi: 10.1016/j.ajpath.2019.08.011 31541644

[165] Glowka TR Steinebach A Stein K Schwandt T Lysson M Holzmann B. The novel CGRP receptor antagonist BIBN4096BS alleviates a postoperative intestinal inflammation and prevents postoperative ileus. Neurogastroenterol Motil (2015) 27:1038–49. doi: 10.1111/nmo.12584 25929169

[166] Ichinose M Sawada M. Enhancement of phagocytosis by calcitonin gene-related peptide (CGRP) in cultured mouse peritoneal macrophages. Peptides (1996) 17:1405–14. doi: 10.1016/s0196-9781(96)00198-2 8971938

[167] Ahmed AA Wahbi AH Nordlin K. Neuropeptides modulate a murine monocyte/macrophage cell line capacity for phagocytosis and killing of leishmania major parasites. Immunopharmacol Immunotoxicol (2001) 23:397–409. doi: 10.1081/iph-100107339 11694030

[168] Ma W Dumont Y Vercauteren F Quirion R. Lipopolysaccharide induces calcitonin gene-related peptide in the RAW264.7 macrophage cell line. Immunology (2010) 130:399–409. doi: 10.1111/j.1365-2567.2009.03239.x 20141542 PMC2913219

[169] Ma W Quirion R. Increased calcitonin gene-related peptide in neuroma and invading macrophages is involved in the up-regulation of interleukin-6 and thermal hyperalgesia in a rat model of mononeuropathy. J Neurochem (2006) 98:180–92. doi: 10.1111/j.1471-4159.2006.03856.x 16805807

[170] Wu R Zhou M Wang P. Adrenomedullin and adrenomedullin binding protein-1 downregulate TNF-alpha in macrophage cell line and rat kupffer cells. Regul Pept (2003) 112:19–26. doi: 10.1016/s0167-0115(03)00018-1 12667621

[171] Wang X Lee CL Vijayan M Yeung WSB Ng EHY Wang X. Adrenomedullin insufficiency alters macrophage activities in fallopian tube: a pathophysiologic explanation of tubal ectopic pregnancy. Mucosal Immunol (2020) 13:743–52. doi: 10.1038/s41385-020-0278-6 32203061

[172] Lv Y Zhang SY Liang X Zhang H Xu Z Liu B. Adrenomedullin 2 enhances beiging in white adipose tissue directly in an adipocyte-autonomous manner and indirectly through activation of M2 macrophages. J Biol Chem (2016) 291:23390–402. doi: 10.1074/jbc.M116.735563 PMC5095396 27621315

[173] Wong LY Cheung BM Li YY Tang F. Adrenomedullin is both proinflammatory and antiinflammatory: its effects on gene expression and secretion of cytokines and macrophage migration inhibitory factor in NR8383 macrophage cell line. Endocrinology (2005) 146:1321–7. doi: 10.1210/en.2004-1080 15576460

[174] Kubo A Minamino N Isumi Y Katafuchi T Kangawa K Dohi K. Production of adrenomedullin in macrophage cell line and peritoneal macrophage. J Biol Chem (1998) 273:16730–8. doi: 10.1074/jbc.273.27.16730 9642228

[175] Nakayama M Takahashi K Murakami O Murakami H Sasano H Shirato K. Adrenomedullin in monocytes and macrophages: possible involvement of macrophage-derived adrenomedullin in atherogenesiS. Clin Sci (Lond) (1999) 97:247–51. 10409481

[176] Hartung HP Toyka KV. Activation of macrophages by substance P: induction of oxidative burst and thromboxane release. Eur J Pharmacol (1983) 89:301–5. doi: 10.1016/0014-2999(83)90511-3 6191998

[177] Yaraee R Ebtekar M Ahmadiani A Sabahi F Ghazanfari T. The effect of substance P on nitric oxide production by HSV-1 infected macrophages. Int Immunopharmacol (2007) 7:135–9. doi: 10.1016/j.intimp.2006.09.001 17178379

[178] Berman AS Chancellor-Freeland C Zhu G Black PH. Substance P primes murine peritoneal macrophages for an augmented proinflammatory cytokine response to lipopolysaccharide. Neuroimmunomodulation (1996) 3:141–9. doi: 10.1159/000097239 8945730

[179] Marriott I Bost KL. Substance P diminishes lipopolysaccharide and interferon-gamma-induced TGF-beta 1 production by cultured murine macrophages. Cell Immunol (1998) 183:113–20. doi: 10.1006/cimm.1998.1248 9606995

[180] Joachim RA Kuhlmei A Dinh QT Handjiski B Fischer T Peters EMJ. Neuronal plasticity of the “brain–skin connection”: stress-triggered up-regulation of neuropeptides in dorsal root ganglia and skin *via* nerve growth factor-dependent pathways. J Mol Med (2007) 85:1369–78. doi: 10.1007/s00109-007-0236-8 17639286

[181] Arck PC Handjiski B Peters EM Peter AS Hagen E Fischer A. Stress inhibits hair growth in mice by induction of premature catagen development and deleterious perifollicular inflammatory events *via* neuropeptide substance P-dependent pathways. Am J Pathol (2003) 162:803–14. doi: 10.1016/S0002-9440(10)63877-1 PMC1868104 12598315

[182] Zhu GF Chancellor-Freeland C Berman AS Kage R Leeman SE Beller DI. Endogenous substance P mediates cold water stress-induced increase in interleukin-6 secretion from peritoneal macrophages. J Neurosci (1996) 16:3745–52. doi: 10.1523/JNEUROSCI.16-11-03745.1996 PMC6578844 8642417

[183] Jiang MH Chung E Chi GF Ahn W Lim JE Hong HS. Substance P induces M2-type macrophages after spinal cord injury. Neuroreport (2012) 23:786–92. doi: 10.1097/WNR.0b013e3283572206 22825006

[184] Lim JE Chung E Son Y. A neuropeptide, Substance-P, directly induces tissue-repairing M2 like macrophages by activating the PI3K/Akt/mTOR pathway even in the presence of IFNgamma. Sci Rep (2017) 7:9417. doi: 10.1038/s41598-017-09639-7 28842601 PMC5573373

[185] Martinez C Delgado M Pozo D Leceta J Calvo JR Ganea D. Vasoactive intestinal peptide and pituitary adenylate cyclase-activating polypeptide modulate endotoxin-induced IL-6 production by murine peritoneal macrophages. J Leukoc Biol (1998) 63:591–601. doi: 10.1002/jlb.63.5.591 9581803

[186] Delgado M Ganea D. Vasoactive intestinal peptide and pituitary adenylate cyclase-activating polypeptide inhibit interleukin-12 transcription by regulating nuclear factor kappaB and Ets activation. J Biol Chem (1999) 274:31930–40. doi: 10.1074/jbc.274.45.31930 10542221

[187] Pozo D Guerrero JM Calvo JR. Vasoactive intestinal peptide and pituitary adenylate cyclase-activating polypeptide inhibit LPS-stimulated MIP-1alpha production and mRNA expression. Cytokine (2002) 18:35–42. doi: 10.1006/cyto.2002.1024 12090758

[188] Delgado M Ganea D. Inhibition of endotoxin-induced macrophage chemokine production by vasoactive intestinal peptide and pituitary adenylate cyclase-activating polypeptide *in vitro* and *in vivo* . J Immunol (2001) 167:966–75. doi: 10.4049/jimmunol.167.2.966 11441105

[189] Delgado M Leceta J Abad C Martinez C Ganea D Gomariz RP. Shedding of membrane-bound CD14 from lipopolysaccharide-stimulated macrophages by vasoactive intestinal peptide and pituitary adenylate cyclase activating polypeptide. J Neuroimmunol (1999) 99:61–71. doi: 10.1016/s0165-5728(99)00105-8 10496178

[190] Delgado M Leceta J Sun W Gomariz RP Ganea D. VIP And PACAP induce shift to a Th2 response by upregulating B7.2 expression. Ann N Y Acad Sci (2000) 921:68–78. doi: 10.1111/j.1749-6632.2000.tb06952.x 11193881

[191] Delgado M Sun W Leceta J Ganea D. VIP and PACAP differentially regulate the costimulatory activity of resting and activated macrophages through the modulation of B7.1 and B7.2 expression. J Immunol (1999) 163:4213–23. doi: 10.4049/jimmunol.163.8.4213 10510358

[192] Kato H Ito A Kawanokuchi J Jin S Mizuno T Ojika K. Pituitary adenylate cyclase-activating polypeptide (PACAP) ameliorates experimental autoimmune encephalomyelitis by suppressing the functions of antigen presenting cells. Mult Scler (2004) 10:651–9. doi: 10.1191/1352458504ms1096oa 15584490

[193] Wada Y Nakamachi T Endo K Seki T Ohtaki H Tsuchikawa D. PACAP attenuates NMDA-induced retinal damage in association with modulation of the microglia/macrophage status into an acquired deactivation subtype. J Mol Neurosci (2013) 51:493–502. doi: 10.1007/s12031-013-0017-5 23720065

[194] Delgado M Garrido E de la Fuente M Gomariz RP. Pituitary adenylate cyclase-activating polypeptide (PACAP-38) stimulates rat peritoneal macrophage functions. Peptides (1996) 17:1097–105. doi: 10.1016/s0196-9781(96)00171-4 8959742

[195] Garrido E Delgado M Martinez C Gomariz RP de la Fuente M. Pituitary adenylate cyclase-activating polypeptide (PACAP38) modulates lymphocyte and macrophage functions: stimulation of adherence and opposite effect on mobility. Neuropeptides (1996) 30:583–95. doi: 10.1016/s0143-4179(96)90042-6 9004257

[196] Sun W Tadmori I Yang L Delgado M Ganea D. Vasoactive intestinal peptide (VIP) inhibits TGF-β1 production in murine macrophages. J Neuroimmunology (2000) 107:88–99. doi: 10.1016/s0165-5728(00)00245-9 10808055

[197] Temerozo JR de Azevedo SSD Insuela DBR Vieira RC Ferreira PLC Carvalho VF. The neuropeptides vasoactive intestinal peptide and pituitary adenylate cyclase-activating polypeptide control HIV-1 infection in macrophages through activation of protein kinases A and C. Front Immunol (2018) 9:1336. doi: 10.3389/fimmu.2018.01336 29951068 PMC6008521

[198] Temerozo JR Joaquim R Regis EG Savino W Bou-Habib DC. Macrophage resistance to HIV-1 infection is enhanced by the neuropeptides VIP and PACAP. PloS One (2013) 8:e67701. doi: 10.1371/journal.pone.0067701 23818986 PMC3688615

[199] Zhu S Hu X Bennett S Mai Y Xu J. Molecular structure, expression and role of TAFA4 and its receptor FPR1 in the spinal cord. Front Cell Dev Biol (2022) 10:911414. doi: 10.3389/fcell.2022.911414 35712659 PMC9194834

[200] Hoeffel G Debroas G Roger A Rossignol R Gouilly J Laprie C. Sensory neuron-derived TAFA4 promotes macrophage tissue repair functions. Nature (2021) 594:94–9. doi: 10.1038/s41586-021-03563-7 34012116

[201] Yang H Zeng Q Silverman HA Gunasekaran M George SJ Devarajan A. HMGB1 released from nociceptors mediates inflammation. Proc Natl Acad Sci U.S.A. (2021). doi: 10.1073/pnas.2102034118 PMC8379951 34385304

[202] Yang H Wang H Andersson U. Targeting inflammation driven by HMGB1. Front Immunol (2020) 11:484. doi: 10.3389/fimmu.2020.00484 32265930 PMC7099994

[203] Nandakumar KS Holmdahl R. Antibody-induced arthritis: disease mechanisms and genes involved at the effector phase of arthritis. Arthritis Res Ther (2006) 8:223. doi: 10.1186/ar2089 17254316 PMC1794524

[204] Kodji X Arkless KL Kee Z Cleary SJ Aubdool AA Evans E. Sensory nerves mediate spontaneous behaviors in addition to inflammation in a murine model of psoriasis. FASEB J (2019) 33:1578–94. doi: 10.1096/fj.201800395RR PMC6338626 30204499

[205] Simeoli R Montague K Jones HR Castaldi L Chambers D Kelleher JH. Exosomal cargo including microRNA regulates sensory neuron to macrophage communication after nerve trauma. Nat Commun (2017) 8:1778. doi: 10.1038/s41467-017-01841-5 29176651 PMC5701122

[206] Liu XJ Liu T Chen G Wang B Yu XL Yin C. TLR signaling adaptor protein MyD88 in primary sensory neurons contributes to persistent inflammatory and neuropathic pain and neuroinflammation. Sci Rep (2016) 6:28188. doi: 10.1038/srep28188 27312666 PMC4911580

[207] Chen O Donnelly CR Ji RR. Regulation of pain by neuro-immune interactions between macrophages and nociceptor sensory neurons. Curr Opin Neurobiol (2020) 62:17–25. doi: 10.1016/j.conb.2019.11.006 31809997 PMC7266706

[208] Guilliams M Mildner A Yona S. Developmental and functional heterogeneity of monocytes. Immunity (2018) 49:595–613. doi: 10.1016/j.immuni.2018.10.005 30332628

[209] Teh YC Ding JL Ng LG Chong SZ. Capturing the fantastic voyage of monocytes through time and space. Front Immunol (2019) 10:834. doi: 10.3389/fimmu.2019.00834 31040854 PMC6476989

[210] Schratzberger P Reinisch N Prodinger WM Kahler CM Sitte BA Bellmann. Differential chemotactic activities of sensory neuropeptides for human peripheral blood mononuclear cells. J Immunol (1997) 158:3895–901. doi: 10.4049/jimmunol.158.8.3895 9103459

[211] Fox FE Kubin M Cassin M Niu Z Hosoi J Torii H. Calcitonin gene-related peptide inhibits proliferation and antigen presentation by human peripheral blood mononuclear cells: effects on B7, interleukin 10, and interleukin 12. J Invest Dermatol (1997) 108:43–8. doi: 10.1111/1523-1747.ep12285627 8980285

[212] Foey AD Field S Ahmed S Jain A Feldmann M Brennan FM. Impact of VIP and cAMP on the regulation of TNF-alpha and IL-10 production: implications for rheumatoid arthritis. Arthritis Res Ther (2003) 5:R317–328. doi: 10.1186/ar999 PMC333423 14680506

[213] Delgado M Ganea D. Vasoactive intestinal peptide inhibits IL-8 production in human monocytes. Biochem Biophys Res Commun (2003) 301:825–32. doi: 10.1016/s0006-291x(03)00059-7 12589787

[214] Lotz M Vaughan JH Carson DA. Effect of neuropeptides on production of inflammatory cytokines by human monocytes. Science (1988) 241:1218–21. doi: 10.1126/science.2457950 2457950

[215] Laurenzi MA Persson MA Dalsgaard CJ Haegerstrand A. The neuropeptide substance P stimulates production of interleukin 1 in human blood monocytes: activated cells are preferentially influenced by the neuropeptide. Scand J Immunol (1990) 31:529–33. doi: 10.1111/j.1365-3083.1990.tb02801.x 1692157

[216] Lee HR Ho WZ Douglas SD. Substance P augments tumor necrosis factor release in human monocyte-derived macrophages. Clin Diagn Lab Immunol (1994) 1:419–23. doi: 10.1128/cdli.1.4.419-423.1994 PMC368279 8556479

[217] Lieb K Fiebich BL Busse-Grawitz M Hull M Berger M Bauer J. Effects of substance P and selected other neuropeptides on the synthesis of interleukin-1 beta and interleukin-6 in human monocytes: a re-examination. J Neuroimmunol (1996) 67:77–81. doi: 10.1016/0165-5728(96)00034-3 8765329

[218] O'Sullivan JA Bochner BS. Eosinophils and eosinophil-associated diseases: An update. J Allergy Clin Immunol (2018) 141:505–17. doi: 10.1016/j.jaci.2017.09.022 PMC5803328 29045815

[219] Prussin G Scott DS Fryer AD Schroeder S Masterson JC Fillon S. Eosinophil cell–cell communication. In: *Eosinophils in health and disease* . Elsevier (2013). 329–90.

[220] Sastre B Rodrigo-Munoz JM Garcia-Sanchez DA Canas JA Del Pozo V. Eosinophils: Old players in a new game. J Investig Allergol Clin Immunol (2018) 28:289–304. doi: 10.18176/jiaci.0295 30059011

[221] Forman R Bramhall M Logunova L Svensson-Frej M Cruickshank SM Else KJ. Eosinophils may play regionally disparate roles in influencing IgA(+) plasma cell numbers during large and small intestinal inflammation. BMC Immunol (2016) 17:12. doi: 10.1186/s12865-016-0153-0 27245920 PMC4886441

[222] Marichal T Mesnil C Bureau F. Homeostatic eosinophils: Characteristics and functions. Front Med (Lausanne) (2017) 4:101. doi: 10.3389/fmed.2017.00101 28744457 PMC5504169

[223] Drake MG Lebold KM Roth-Carter QR Pincus AB Blum ED Proskocil BJ. Eosinophil and airway nerve interactions in asthma. J Leukoc Biol (2018) 104:61–7. doi: 10.1002/JLB.3MR1117-426R PMC6541210 29633324

[224] Liang Y Jacobi HH Reimert CM Haak-Frendscho M Marcusson JA Johansson O. CGRP-immunoreactive nerves in prurigo nodularis–an exploration of neurogenic inflammation. J Cutan Pathol (2000) 27:359–66. doi: 10.1034/j.1600-0560.2000.027007359.x 10917163

[225] Guseva D Rudrich U Kotnik N Gehring M Patsinakidis N Agelopoulos K. Neuronal branching of sensory neurons is associated with BDNF-positive eosinophils in atopic dermatitis. Clin Exp Allergy (2020) 50:577–84. doi: 10.1111/cea.13560 31925827

[226] O'Brien LM Fitzpatrick E Baird AW Campion DP. Eosinophil-nerve interactions and neuronal plasticity in rat gut associated lymphoid tissue (GALT) in response to enteric parasitism. J Neuroimmunol (2008) 197:1–9. doi: 10.1016/j.jneuroim.2008.04.002 18495257

[227] Foster EL Simpson EL Fredrikson LJ Lee JJ Lee NA Fryer AD. Eosinophils increase neuron branching in human and murine skin and *in vitro* . PloS One (2011) 6:e22029. doi: 10.1371/journal.pone.0022029 21811556 PMC3140999

[228] Pelaquini EH Guimaraes Lde A Benetti LR Fernandes LG Tamashiro WM Conran N. Role of the Mac-1 and VLA-4 integrins, and concomitant Th2-cytokine production, in nitric oxide modulated eosinophil migration from bone marrow to lungs in allergic mice. Int Immunopharmacol (2011) 11:204–11. doi: 10.1016/j.intimp.2010.11.020 21111080

[229] Dunzendorfer S Feistritzer C Enrich B Wiedermann CJ. Neuropeptide-induced inhibition of IL-16 release from eosinophils. Neuroimmunomodulation (2002) 10:217–23. doi: 10.1159/000068324 12584409

[230] Kroegel C Giembycz MA Barnes PJ. Characterization of eosinophil cell activation by peptides. differential effects of substance P, melittin, and FMET-Leu-Phe. J Immunol (1990) 145:2581–7. doi: 10.4049/jimmunol.145.8.2581 1698858

[231] Acharya KR Ackerman SJ. Eosinophil granule proteins: form and function. J Biol Chem (2014) 289:17406–15. doi: 10.1074/jbc.R113.546218 PMC4067173 24802755

[232] Iwamoto I Nakagawa N Yamazaki H Kimura A Tomioka H Yoshida S. Mechanism for substance P-induced activation of human neutrophils and eosinophils. Regul Pept (1993) 46:228–30. doi: 10.1016/0167-0115(93)90042-7 7692498

[233] Raap M Rudrich U Stander S Gehring M Kapp A Raap U. Substance P activates human eosinophils. Exp Dermatol (2015) 24:557–9. doi: 10.1111/exd.12717 25865137

[234] Foster AP Cunningham FM. Substance P induces activation, adherence and migration of equine eosinophils. J Vet Pharmacol Ther (2003) 26:131–8. doi: 10.1046/j.1365-2885.2003.00453.x 12667183

[235] Smith CH Barker JN Morris RW MacDonald DM Lee TH. Neuropeptides induce rapid expression of endothelial cell adhesion molecules and elicit granulocytic infiltration in human skin. J Immunol (1993) 151:3274–82. doi: 10.4049/jimmunol.151.6.3274 7690800

[236] Baluk P Bertrand C Geppetti P McDonald DM Nadel JA. NK1 receptors mediate leukocyte adhesion in neurogenic inflammation in the rat trachea. Am J Physiol (1995) 268:L263–269. doi: 10.1152/ajplung.1995.268.2.L263 7864147

[237] Numao T Agrawal DK. Neuropeptides modulate human eosinophil chemotaxis. J Immunol (1992) 149:3309–15. doi: 10.4049/jimmunol.149.10.3309 1385521

[238] Fajac I Braunstein G Ickovic MR Lacronique J Frossard N. Selective recruitment of eosinophils by substance P after repeated allergen exposure in allergic rhinitis. Allergy (1995) 50:970–5. doi: 10.1111/j.1398-9995.1995.tb02509.x 8834826

[239] Mehta D Granstein RD. Immunoregulatory effects of neuropeptides on endothelial cells: Relevance to dermatological disorders. Dermatology (2019) 235:175–86. doi: 10.1159/000496538 30808842

[240] El-Shazly AE Masuyama K Eura M Ishikawa T. Immunoregulatory effect of substance P in human eosinophil migratory function. Immunol Invest (1996) 25:191–201. doi: 10.3109/08820139609059302 9157054

[241] Dunzendorfer S Meierhofer C Wiedermann CJ. Signaling in neuropeptide-induced migration of human eosinophils. J Leukoc Biol (1998) 64:828–34. doi: 10.1002/jlb.64.6.828 9850167

[242] Caceres AI Brackmann M Elia MD Bessac BF del Camino D D'Amours M. A sensory neuronal ion channel essential for airway inflammation and hyperreactivity in asthma. Proc Natl Acad Sci U.S.A. (2009) 106:9099–104. doi: 10.1073/pnas.0900591106 PMC2684498 19458046

[243] Trankner D Hahne N Sugino K Hoon MA Zuker C. Population of sensory neurons essential for asthmatic hyperreactivity of inflamed airways. Proc Natl Acad Sci USA (2014) 111:11515–20. doi: 10.1073/pnas.1411032111 PMC4128113 25049382

[244] Evans CM Belmonte KE Costello RW Jacoby DB Gleich GJ Fryer AD. Substance P-induced airway hyperreactivity is mediated by neuronal M2 receptor dysfunction. Am J Physiol Lung Cell Mol Physiol (2000) 279:L477–86. doi: 10.1152/ajplung.2000.279.3.L477 10956622

[245] Balestrini A Joseph V Dourado M Reese RM Shields SD Rouge L. A TRPA1 inhibitor suppresses neurogenic inflammation and airway contraction for asthma treatment. J Exp Med (2021) 218. doi: 10.1084/jem.20201637 PMC7918756 33620419

[246] Chirumbolo S Bjorklund G Sboarina A Vella A. The role of basophils as innate immune regulatory cells in allergy and immunotherapy. Hum Vaccin Immunother (2018) 14:815–31. doi: 10.1080/21645515.2017.1417711 PMC5893214 29257936

[247] Siracusa MC Kim BS Spergel JM Artis D. Basophils and allergic inflammation. J Allergy Clin Immunol (2013) 132:789–801. doi: 10.1016/j.jaci.2013.07.046 24075190 PMC3903395

[248] Otsuka A Kabashima K. Contribution of basophils to cutaneous immune reactions and Th2-mediated allergic responses. Front Immunol (2015) 6:393. doi: 10.3389/fimmu.2015.00393 26284076 PMC4522869

[249] Cima K Vogelsinger H Kahler CM. Sensory neuropeptides are potent chemoattractants for human basophils *in vitro* . Regul Pept (2010) 160:42–8. doi: 10.1016/j.regpep.2009.12.013 20035805

[250] Zheng W Wang J Zhu W Xu C He S. Upregulated expression of substance P in basophils of the patients with chronic spontaneous urticaria: Induction of histamine release and basophil accumulation by substance p. Cell Biol Toxicol (2016) 32:217–28. doi: 10.1007/s10565-016-9330-4 27147256

[251] Nakashima C Ishida Y Kitoh A Otsuka A Kabashima K. Interaction of peripheral nerves and mast cells, eosinophils, and basophils in the development of pruritus. Exp Dermatol (2019) 28:1405–11. doi: 10.1111/exd.14014 31365150

[252] Liew PX Kubes P. The neutrophil's role during health and disease. Physiol Rev (2019) 99:1223–48. doi: 10.1152/physrev.00012.2018 30758246

[253] Kolaczkowska E Jenne CN Surewaard BG Thanabalasuriar A Lee WY Sanz MJ. Molecular mechanisms of NET formation and degradation revealed by intravital imaging in the liver vasculature. Nat Commun (2015) 6:6673. doi: 10.1038/ncomms7673 25809117 PMC4389265

[254] Khan AA Alsahli MA Rahmani AH. Myeloperoxidase as an active disease biomarker: recent biochemical and pathological perspectives. Med Sci (Basel) (2018) 6. doi: 10.3390/medsci6020033 PMC6024665 29669993

[255] Strausbaugh HJ Green PG Lo E Tangemann K Reichling DB Rosen SD. Painful stimulation suppresses joint inflammation by inducing shedding of l-selectin from neutrophils. Nat Med (1999) 5:1057–61. doi: 10.1038/12497 10470085

[256] Ley K Laudanna C Cybulsky MI Nourshargh S. Getting to the site of inflammation: the leukocyte adhesion cascade updated. Nat Rev Immunol (2007) 7:678–89. doi: 10.1038/nri2156 17717539

[257] Elekes K Helyes Z Nemeth J Sandor K Pozsgai G Kereskai L. Role of capsaicin-sensitive afferents and sensory neuropeptides in endotoxin-induced airway inflammation and consequent bronchial hyperreactivity in the mouse. Regul Pept (2007) 141:44–54. doi: 10.1016/j.regpep.2006.12.018 17291600

[258] Kihara N de la Fuente SG Fujino K Takahashi T Pappas TN Mantyh CR. Vanilloid receptor-1 containing primary sensory neurones mediate dextran sulphate sodium induced colitis in rats. Gut (2003) 52:713–9. doi: 10.1136/gut.52.5.713 PMC1773638 12692058

[259] Fujino K Takami Y de la Fuente SG Ludwig KA Mantyh CR. Inhibition of the vanilloid receptor subtype-1 attenuates TNBS-colitis. J Gastrointest Surg (2004) 8:842–7. doi: 10.1016/j.gassur.2004.07.011 15531237

[260] Miranda A Nordstrom E Mannem A Smith C Banerjee B Sengupta JN. The role of transient receptor potential vanilloid 1 in mechanical and chemical visceral hyperalgesia following experimental colitis. Neuroscience (2007) 148:1021–32. doi: 10.1016/j.neuroscience.2007.05.034 PMC2128774 17719181

[261] Okayama M Tsubouchi R Kato S Takeuchi K. Protective effect of lafutidine, a novel histamine H2-receptor antagonist, on dextran sulfate sodium-induced colonic inflammation through capsaicin-sensitive afferent neurons in rats. Diges Dis Sci (2004) 49:1696–704. doi: 10.1023/b:ddas.0000043389.96490.76 15573930

[262] Sanz-Salvador L Andres-Borderia A Ferrer-Montiel A Planells-Cases R. Agonist- and Ca2+-dependent desensitization of TRPV1 channel targets the receptor to lysosomes for degradation. J Biol Chem (2012) 287:19462–71. doi: 10.1074/jbc.M111.289751 PMC3365984 22493457

[263] Monneret G Arpin M Venet F Maghni K Debard AL Pachot A. Calcitonin gene related peptide and N-procalcitonin modulate CD11b upregulation in lipopolysaccharide activated monocytes and neutrophils. Intensive Care Med (2003) 29:923–8. doi: 10.1007/s00134-003-1759-2 12712241

[264] Jochheim LS Odysseos G Hidalgo-Sastre A Zhong S Staufer LM Kroiss M. The neuropeptide receptor subunit RAMP1 constrains the innate immune response during acute pancreatitis in mice. Pancreatology (2019) 19:541–7. doi: 10.1016/j.pan.2019.05.455 31109903

[265] Jusek G Reim D Tsujikawa K Holzmann B. Deficiency of the CGRP receptor component RAMP1 attenuates immunosuppression during the early phase of septic peritonitis. Immunobiology (2012) 217:761–7. doi: 10.1016/j.imbio.2012.04.009 22656887

[266] Lei J Zhu F Zhang Y Duan L Lei H Huang W. Transient receptor potential vanilloid subtype 1 inhibits inflammation and apoptosis *via* the release of calcitonin gene-related peptide in the heart after myocardial infarction. Cardiology (2016) 134:436–43. doi: 10.1159/000444439 27144592

[267] Gao Z Liu Y Zhang L Yang Z Lv L Wang S. Nociceptor neurons are involved in the host response to Escherichia coli urinary tract infections. J Inflammation Res (2022) 15:3337–53. doi: 10.2147/JIR.S356960 PMC9188809 35702548

[268] Serra MC Bazzoni F Della Bianca V Greskowiak M Rossi F. Activation of human neutrophils by substance P. Effect on oxidative metabolism, excocytosis, cytosolic Ca2+ concentration and inositol phosphate formation. J Immunol (1988) 141:2118–24. doi: 10.4049/jimmunol.141.6.2118 2459200

[269] Bockmann S Seep J Jonas L. Delay of neutrophil apoptosis by the neuropeptide substance P: involvement of caspase cascade. Peptides (2001) 22:661–70. doi: 10.1016/s0196-9781(01)00376-x 11311737

[270] Ohlen A Thureson-Klein A Lindbom L Persson MG Hedqvist P. Substance P activates leukocytes and platelets in rabbit microvessels. Blood Vessels (1989) 26:84–94. doi: 10.1159/000158757 2474341

[271] Wozniak A McLennan G Betts WH Murphy GA Scicchitano R. Activation of human neutrophils by substance P: effect on FMLP-stimulated oxidative and arachidonic acid metabolism and on antibody-dependent cell-mediated cytotoxicity. Immunology (1989) 68:359–64. PMC1385448 2480329

[272] Martinez C Juarranz Y Abad C Arranz A Miguel BG Rosignoli F. Analysis of the role of the PAC1 receptor in neutrophil recruitment, acute-phase response, and nitric oxide production in septic shock. J Leukoc Biol (2005) 77:729–38. doi: 10.1189/jlb.0704432 15661828

[273] Sergejeva S Hoshino H Yoshihara S Kashimoto K Lotvall J Linden A. A synthetic VIP peptide analogue inhibits neutrophil recruitment in rat airways *in vivo* . Regul Pept (2004) 117:149–54. doi: 10.1016/j.regpep.2003.10.002 14700751

[274] Li C Liu YY Zhao GQ Lin J Che CY Jiang N. Role of vasoactive intestinal peptide in aspergillus fumigatus-infected cornea. Int J Ophthalmol (2018) 11:183–8. doi: 10.18240/ijo.2018.02.01 PMC5824069 29487804

[275] Stead RH Dixon MF Bramwell NH Riddell RH Bienenstock J. Mast cells are closely apposed to nerves in the human gastrointestinal mucosa. Gastroenterology (1989) 97:575–85. doi: 10.1016/0016-5085(89)90627-6 2666250

[276] Kilinc E Guerrero-Toro C Zakharov A Vitale C Gubert-Olive M Koroleva K. Serotonergic mechanisms of trigeminal meningeal nociception: Implications for migraine pain. Neuropharmacology (2017) 116:160–73. doi: 10.1016/j.neuropharm.2016.12.024 28025094

[277] Balemans D Aguilera-Lizarraga J Florens MV Jain P Denadai-Souza A Viola MF. Histamine-mediated potentiation of transient receptor potential (TRP) ankyrin 1 and TRP vanilloid 4 signaling in submucosal neurons in patients with irritable bowel syndrome. Am J Physiol Gastrointest Liver Physiol (2019) 316:G338–49. doi: 10.1152/ajpgi.00116.2018 30629470

[278] Wernersson S Pejler G. Mast cell secretory granules: armed for battle. Nat Rev Immunol (2014) 14:478–94. doi: 10.1038/nri3690 24903914

[279] Gupta K Harvima IT. Mast cell-neural interactions contribute to pain and itch. Immunol Rev (2018) 282:168–87. doi: 10.1111/imr.12622 PMC5812374 29431216

[280] Gaudenzio N Sibilano R Marichal T Starkl P Reber LL Cenac N. Different activation signals induce distinct mast cell degranulation strategies. J Clin Invest (2016) 126:3981–98. doi: 10.1172/JCI85538 PMC5096814 27643442

[281] Kunder CA St John AL Li G Leong KW Berwin B Staats HF. Mast cell-derived particles deliver peripheral signals to remote lymph nodes. J Exp Med (2009) 206:2455–67. doi: 10.1084/jem.20090805 PMC2768851 19808250

[282] Kulka M Sheen CH Tancowny BP Grammer LC Schleimer RP. Neuropeptides activate human mast cell degranulation and chemokine production. Immunology (2008) 123:398–410. doi: 10.1111/j.1365-2567.2007.02705.x 17922833 PMC2433325

[283] Xing W Austen KF Gurish MF Jones TG. Protease phenotype of constitutive connective tissue and of induced mucosal mast cells in mice is regulated by the tissue. Proc Natl Acad Sci U.S.A. (2011) 108:14210–5. doi: 10.1073/pnas.1111048108 PMC3161524 21825171

[284] Gilfillan AM Beaven MA. Regulation of mast cell responses in health and disease. Crit Rev Immunol (2011) 31:475–529. doi: 10.1615/critrevimmunol.v31.i6.30 22321108 PMC3395887

[285] Pundir P Liu R Vasavda C Serhan N Limjunyawong N Yee R. A connective tissue mast-cell-specific receptor detects bacterial quorum-sensing molecules and mediates antibacterial immunity. Cell Host Microbe (2019) 26:114–122.e118. doi: 10.1016/j.chom.2019.06.003 31278040 PMC6649664

[286] Musch W Wege AK Mannel DN Hehlgans T. Generation and characterization of alpha-chymase-Cre transgenic mice. Genesis (2008) 46:163–6. doi: 10.1002/dvg.20378 18327770

[287] Dudeck A Dudeck J Scholten J Petzold A Surianarayanan S Kohler A. Mast cells are key promoters of contact allergy that mediate the adjuvant effects of haptens. Immunity (2011) 34:973–84. doi: 10.1016/j.immuni.2011.03.028 21703544

[288] Li Z Liu S Xu J Zhang X Han D Liu J. Adult connective tissue-resident mast cells originate from late erythro-myeloid progenitors. Immunity (2018) 49:640–653 e645. doi: 10.1016/j.immuni.2018.09.023 30332630

[289] Hsieh FH Sharma P Gibbons A Goggans T Erzurum SC Haque SJ. Human airway epithelial cell determinants of survival and functional phenotype for primary human mast cells. Proc Natl Acad Sci U.S.A. (2005) 102:14380–5. doi: 10.1073/pnas.0503948102 PMC1242292 16186496

[290] Mierke CT Ballmaier M Werner U Manns MP Welte K Bischoff SC. Human endothelial cells regulate survival and proliferation of human mast cells. J Exp Med (2000) 192:801–11. doi: 10.1084/jem.192.6.801 PMC2193280 10993911

[291] Le DD Schmit D Heck S Omlor AJ Sester M Herr C. Increase of mast cell-nerve association and neuropeptide receptor expression on mast cells in perennial allergic rhinitis. Neuroimmunomodulation (2016) 23:261–70. doi: 10.1159/000453068 28030866

[292] Levick SP Brower GL Janicki JS. Substance P-mediated cardiac mast cell activation: An *in vitro* study. Neuropeptides (2019) 74:52–9. doi: 10.1016/j.npep.2019.01.002 PMC7207245 30660328

[293] Zhan M Zheng W Jiang Q Zhao Z Wang Z Wang J. Upregulated expression of substance P (SP) and NK1R in eczema and SP-induced mast cell accumulation. Cell Biol Toxicol (2017) 33:389–405. doi: 10.1007/s10565-016-9379-0 28154998

[294] Meixiong J Basso L Dong X Gaudenzio N. Nociceptor-mast cell sensory clusters as regulators of skin homeostasis. Trends Neurosci (2020) 43:130–2. doi: 10.1016/j.tins.2020.01.001 32014258

[295] Caughey GH Leidig F Viro NF Nadel JA. Substance P and vasoactive intestinal peptide degradation by mast cell tryptase and chymase. J Pharmacol Exp Ther (1988) 244:133–7. 2447273

[296] Arifuzzaman M Mobley YR Choi HW Bist P Salinas CA Brown ZD. MRGPR-mediated activation of local mast cells clears cutaneous bacterial infection and protects against reinfection. Sci Adv (2019) 5:eaav0216. doi: 10.1126/sciadv.aav0216 30613778 PMC6314830

[297] Tancowny BP Karpov V Schleimer RP Kulka M. Substance P primes lipoteichoic acid- and Pam3CysSerLys4-mediated activation of human mast cells by up-regulating Toll-like receptor 2. Immunology (2010) 131:220–30. doi: 10.1111/j.1365-2567.2010.03296.x PMC2967268 20497485

[298] Zhang S Edwards TN Chaudhri VK Wu J Cohen JA Hirai T. Nonpeptidergic neurons suppress mast cells *via* glutamate to maintain skin homeostasis. Cell (2021) 184:2151–2166.e2116. doi: 10.1016/j.cell.2021.03.002 33765440 PMC8052305

[299] Azzolina A Bongiovanni A Lampiasi N. Substance P induces TNF-alpha and IL-6 production through NF kappa B in peritoneal mast cells. Biochim Biophys Acta (2003) 1643:75–83. doi: 10.1016/j.bbamcr.2003.09.003 14654230

[300] Piotrowski W Foreman JC. On the actions of substance P, somatostatin, and vasoactive intestinal polypeptide on rat peritoneal mast cells and in human skin. Naunyn Schmiedebergs Arch Pharmacol (1985) 331:364–8. doi: 10.1007/BF00500821 2419771

[301] Manning BM Gruba SM Meyer AF Haynes CL. Neuropeptide-induced mast cell degranulation and characterization of signaling modulation in response to IgE conditioning. ACS Chem Biol (2016) 11:3077–83. doi: 10.1021/acschembio.6b00616 27580075

[302] Ansel JC Brown JR Payan DG Brown MA. Substance P selectively activates TNF-alpha gene expression in murine mast cells. J Immunol (1993) 150:4478–85. doi: 10.4049/jimmunol.150.10.4478 7683320

[303] Piotrowski W Foreman JC. Some effects of calcitonin gene-related peptide in human skin and on histamine release. Br J Dermatol (1986) 114:37–46. doi: 10.1111/j.1365-2133.1986.tb02777.x 2417614

[304] Niizeki H Alard P Streilein JW. Calcitonin gene-related peptide is necessary for ultraviolet B-impaired induction of contact hypersensitivity. J Immunol (1997) 159:5183–6. doi: 10.4049/jimmunol.159.11.5183 9548453

[305] Zudaire E Martinez A Garayoa M Pio R Kaur G Woolhiser MR. Adrenomedullin is a cross-talk molecule that regulates tumor and mast cell function during human carcinogenesis. Am J Pathol (2006) 168:280–91. doi: 10.2353/ajpath.2006.050291 PMC1592665 16400030

[306] Lv YP Peng LS Wang QH Chen N Teng YS Wang TT. Degranulation of mast cells induced by gastric cancer-derived adrenomedullin prompts gastric cancer progression. Cell Death Dis (2018) 9:1034. doi: 10.1038/s41419-018-1100-1 30305610 PMC6180028

[307] Uzuner K Tuncel N Aydin Y Tuncel M Gurer F Benli P. The effect of vasoactive intestinal peptide (VIP) on superoxide dismutase and catalase activities in renal tissues of rats exposed to hemorrhagic ischemia-reperfusion. Peptides (1995) 16:911–5. doi: 10.1016/0196-9781(95)00055-o 7479334

[308] Stakenborg N Viola MF Boeckxstaens GE. Intestinal neuro-immune interactions: focus on macrophages, mast cells and innate lymphoid cells. Curr Opin Neurobiol (2020) 62:68–75. doi: 10.1016/j.conb.2019.11.020 31862627 PMC7294228

[309] Bischoff SC Schwengberg S Lorentz A Manns MP Bektas H Sann H. Substance P and other neuropeptides do not induce mediator release in isolated human intestinal mast cells. Neurogastroenterol Motil (2004) 16:185–93. doi: 10.1111/j.1365-2982.2004.00502.x 15086872

[310] Wang GD Wang XY Liu S Qu M Xia Y Needleman BJ. Innervation of enteric mast cells by primary spinal afferents in guinea pig and human small intestine. Am J Physiol Gastrointest Liver Physiol (2014) 307:G719–731. doi: 10.1152/ajpgi.00125.2014 PMC4187066 25147231

[311] Keita AV Carlsson AH Cigehn M Ericson AC McKay DM Soderholm JD. Vasoactive intestinal polypeptide regulates barrier function *via* mast cells in human intestinal follicle-associated epithelium and during stress in rats. Neurogastroenterol Motil (2013) 25:e406–417. doi: 10.1111/nmo.12127 23600853

[312] Lee J Yamamoto T Hayashi S Kuramoto H Kadowaki M. Enhancement of CGRP sensory afferent innervation in the gut during the development of food allergy in an experimental murine model. Biochem Biophys Res Commun (2013) 430:895–900. doi: 10.1016/j.bbrc.2012.12.058 23261435

[313] De Jonge F Van Nassauw L Adriaensen D Van Meir F Miller HR Van Marck E. Effect of intestinal inflammation on capsaicin-sensitive afferents in the ileum of schistosoma mansoni-infected mice. Histochem Cell Biol (2003) 119:477–84. doi: 10.1007/s00418-003-0532-5 12768286

[314] Barbara G Stanghellini V De Giorgio R Cremon C Cottrell GS Santini D. Activated mast cells in proximity to colonic nerves correlate with abdominal pain in irritable bowel syndrome. Gastroenterology (2004) 126:693–702. doi: 10.1053/j.gastro.2003.11.055 14988823

[315] Saban R Gerard NP Saban MR Nguyen NB DeBoer DJ Wershil BK. Mast cells mediate substance P-induced bladder inflammation through an NK(1) receptor-independent mechanism. Am J Physiol Renal Physiol (2002) 283:F616–629. doi: 10.1152/ajprenal.00096.2002 12217852

[316] Andersson CK Mori M Bjermer L Lofdahl CG Erjefalt JS. Novel site-specific mast cell subpopulations in the human lung. Thorax (2009) 64:297–305. doi: 10.1136/thx.2008.101683 19131451

[317] Varricchi G Pecoraro A Loffredo S Poto R Rivellese F Genovese A. Heterogeneity of human mast cells with respect to MRGPRX2 receptor expression and function. Front Cell Neurosci (2019) 13:299. doi: 10.3389/fncel.2019.00299 31333418 PMC6616107

[318] Manorak W Idahosa C Gupta K Roy S Panettieri R Jr Ali H. Upregulation of mas-related G protein coupled receptor X2 in asthmatic lung mast cells and its activation by the novel neuropeptide hemokinin-1. Respir Res (2018) 19:1. doi: 10.1186/s12931-017-0698-3 29295703 PMC5751818

[319] Nieber K Baumgarten CR Rathsack R Furkert J Oehme P Kunkel G. Substance P and beta-endorphin-like immunoreactivity in lavage fluids of subjects with and without allergic asthma. J Allergy Clin Immunol (1992) 90:646–52. doi: 10.1016/0091-6749(92)90138-r 1383307

[320] Forsythe P McGarvey LP Heaney LG MacMahon J Ennis M. Sensory neuropeptides induce histamine release from bronchoalveolar lavage cells in both nonasthmatic coughers and cough variant asthmatics. Clin Exp Allergy (2000) 30:225–32. doi: 10.1046/j.1365-2222.2000.00770.x 10651775

[321] Rozniecki JJ Dimitriadou V Lambracht-Hall M Pang X Theoharides TC. Morphological and functional demonstration of rat dura mater mast cell-neuron interactions *in vitro* and *in vivo* . Brain Res (1999) 849:1–15. doi: 10.1016/s0006-8993(99)01855-7 10592282

[322] Ottosson A Edvinsson L. Release of histamine from dural mast cells by substance P and calcitonin gene-related peptide. Cephalalgia (1997) 17:166–74. doi: 10.1046/j.1468-2982.1997.1703166.x 9170339

[323] Goadsby PJ Edvinsson L. The trigeminovascular system and migraine: studies characterizing cerebrovascular and neuropeptide changes seen in humans and cats. Ann Neurol (1993) 33:48–56. doi: 10.1002/ana.410330109 8388188

[324] Bree D Levy D. Development of CGRP-dependent pain and headache related behaviours in a rat model of concussion: Implications for mechanisms of post-traumatic headache. Cephalalgia (2018) 38:246–58. doi: 10.1177/0333102416681571 PMC6454269 27899434

[325] Yan LY Dong X Xue LJ Xu H Zhou ZK Wan Q. Neurogenic dural inflammation induced by inflammatory soup combined with CGRP: a modified animal model of migraine. Int J Clin Exp Med (2018) 11:9126–34.

[326] Eftekhari S Warfvinge K Blixt FW Edvinsson L. Differentiation of nerve fibers storing CGRP and CGRP receptors in the peripheral trigeminovascular system. J Pain (2013) 14:1289–303. doi: 10.1016/j.jpain.2013.03.010 23958278

[327] Tuncel N Sener E Cerit C Karasu U Gurer F Sahinturk V. Brain mast cells and therapeutic potential of vasoactive intestinal peptide in a parkinson's disease model in rats: brain microdialysis, behavior, and microscopy. Peptides (2005) 26:827–36. doi: 10.1016/j.peptides.2004.12.019 15808913

[328] Malaviya R Ikeda T Ross E Abraham SN. Mast cell modulation of neutrophil influx and bacterial clearance at sites of infection through TNF-alpha. Nature (1996) 381:77–80. doi: 10.1038/381077a0 8609993

[329] Svensson E Apergis-Schoute J Burnstock G Nusbaum MP Parker D Schioth HB. General principles of neuronal co-transmission: insights from multiple model systems. Front Neural Circuits (2018) 12:117. doi: 10.3389/fncir.2018.00117 30728768 PMC6352749

[330] Nusbaum MP Blitz DM Marder E. Functional consequences of neuropeptide and small-molecule co-transmission. Nat Rev Neurosci (2017) 18:389–403. doi: 10.1038/nrn.2017.56 28592905 PMC5547741

[331] MacLean DB Bennett B Morris M Wheeler FB. Differential regulation of calcitonin ene-related peptide and substance P in cultured neonatal rat vagal sensory neurons. Brain Res (1989) 478:349–55. doi: 10.1016/0006-8993(89)91515-1 2466532

[332] Finger TE. Peptide immunohistochemistry demonstrates multiple classes of perigemmal nerve fibers in the circumvallate papilla of the rat. Chem Senses (1986) 11:135–44. doi: 10.1093/chemse/11.1.135

[333] Hsieh YL Lin CL Chiang H Fu YS Lue JH Hsieh ST. Role of peptidergic nerve terminals in the skin: reversal of thermal sensation by calcitonin gene-related peptide in TRPV1-depleted neuropathy. PloS One (2012) 7:e50805. doi: 10.1371/journal.pone.0050805 23209829 PMC3507736

[334] Russo AF. Overview of neuropeptides: Awakening the senses? Headache (2017) 57 Suppl 2:37–46. doi: 10.1111/head.13084 28485842 PMC5424629

[335] Hokfelt T Johansson O Ljungdahl A Lundberg JM Schultzberg M. Peptidergic neurones. Nature (1980) 284:515–21. doi: 10.1038/284515a0 6154244

[336] Toth CC Willis D Twiss JL Walsh S Martinez JA Liu WQ. Locally synthesized calcitonin gene-related peptide has a critical role in peripheral nerve regeneration. J Neuropathol Exp Neurol (2009) 68:326–37. doi: 10.1097/NEN.0b013e31819ac71b PMC5790422 19225405

[337] Biever A Donlin-Asp PG Schuman EM. Local translation in neuronal processes. Curr Opin Neurobiol (2019) 57:141–8. doi: 10.1016/j.conb.2019.02.008 30861464

[338] Obara I Geranton SM Hunt SP. Axonal protein synthesis: a potential target for pain relief? Curr Opin Pharmacol (2012) 12:42–8. doi: 10.1016/j.coph.2011.10.005 22033338

[339] Baral P Udit S Chiu IM. Pain and immunity: implications for host defence. Nat Rev Immunol (2019) 19:433–47. doi: 10.1038/s41577-019-0147-2 PMC6700742 30874629

[340] Pinho-Ribeiro FA Verri WA Jr Chiu IM. Nociceptor sensory neuron-immune interactions in pain and inflammation. Trends Immunol (2017) 38:5–19. doi: 10.1016/j.it.2016.10.001 27793571 PMC5205568

